# Sulfur source-driven global remodeling of gene expression in the fuel-biodesulfurizing *Rhodococcus qingshengii* IGTS8

**DOI:** 10.1128/aem.01549-26

**Published:** 2026-09-23

**Authors:** Ashraf El Nayal, Vartul Sangal, Abdelmalek Alioua, Aurélie Hirschler, Sandrine Koechler, Dimitri Heintz, Christine Carapito, Alain Van Dorsselaer, Wael Ismail

**Affiliations:** 1 Center of Environmental and Biological Studies, Arabian Gulf University 108540, Manama, Bahrain; 2 School of Geography and Natural Sciences, Northumbria University 5995, Newcastle upon Tyne, United Kingdom; 3 Institut de biologie de moléculaire des plantes CNRS, Strasbourg, France; 4 Laboratoire de Spectrométrie de Masse BioOrganique, CNRS, Université de Strasbourg, IPHC UMR 7178 27083, Strasbourg, France; 5 Université de Strasbourg, CNRS, IPHC UMR 7178, Strasbourg, France; University of Nebraska-Lincoln, Lincoln, Nebraska, USA

**Keywords:** mycofactocin, sulfur metabolism, stress response, transcriptomics, dibenzothiophene, 4S pathway

## Abstract

Recent proteomics and metabolomics studies have confirmed the significance of understanding the fuel biodesulfurization phenotype at a global scale beyond the desulfurization 4S pathway. To reveal the transcriptomic adaptations in the model strain *Rhodococcus qingshengii* IGTS8 under biodesulfurization conditions, we conducted a time series comparative RNA-seq study using cultures of IGTS8 grown on either dibenzothiophene (DBT) or MgSO_4_ as the sulfur source. The RNA-seq data revealed many differentially expressed genes (DEGs) depending on the sulfur source and growth phase, and the strongest transcriptomic response was evident during the late-log phase. In total, 6,690 transcripts were detected in the DBT and MgSO_4_ cultures, representing 99.5% of the total protein-coding genes. A substantial fraction of the DEGs encodes ABC-type transporters and hypothetical proteins. In the DBT culture, 623 DEGs were upregulated, and 587 DEGs were downregulated. Generally, the DEGs covered key physiological processes, such as stress response, regulation of gene expression, uptake of nutrients, metabolism of lipids, amino acids, carbohydrates, proteins, nucleotides, cell division, and cell wall biosynthesis/degradation. While genes of carbohydrate metabolism were mostly downregulated in the DBT culture, those encoding amino acid and fatty acid degradation, as well as energy conservation, were upregulated. Moreover, genes encoding mobile genetic elements, C1 metabolism enzymes, carbonic anhydrase, and nitrile hydratase were upregulated under biodesulfurization conditions. In contrast, dihydrofolate reductase was strongly downregulated. The growth phase-dependent transcriptomic rearrangements under biodesulfurization conditions appear to prioritize cellular homeostasis and protection rather than proliferation.

**IMPORTANCE:**

Commercial application of fuel biodesulfurization requires exceptionally robust and catalytically active desulfurizing microbes that can tolerate and sustain activity under the harsh operational conditions in oil refineries. It is generally accepted that naturally occurring bacteria may not meet these industrial requirements. Therefore, synthetic biology and metabolic engineering hold promise. However, a thorough understanding of the physiology, metabolism, and stress response of fuel-desulfurizing bacteria, which is currently lacking, is key toward further developments in this field. Considering these knowledge gaps, our findings provide mechanistic insights into the transcriptional responses of the reference biodesulfurizing strain *Rhodococcus qingshengii* IGTS8, which, when integrated together with our recently reported proteomics and metabolomics data, enable a multifaceted and deeper understanding of the biodesulfurization phenotype, facilitating rational design of more efficient recombinant biodesulfurizers. Hence, this study represents a step forward toward developing a commercially viable fuel biodesulfurization process.

## INTRODUCTION

Fossil fuels contain various organosulfur compounds, including dibenzothiophene (DBT) and its alkyl derivatives, which, upon combustion, release environmentally hazardous sulfur dioxide. In addition, some fuel-borne organosulfur compounds are harmful to public health (1–3). Hence, strict environmental regulations mandate a drastic reduction in the sulfur content of transportation fuels to avoid environmental pollution and meet globally increasing demands for cleaner fuels (4, 5). Oil refineries routinely apply a thermochemical treatment, namely hydrodesulfurization, to remove sulfur from crude oil and diesel. This conventional treatment is associated with economic, technical, and environmental shortcomings, which have prompted the need for green, cost-effective, and more efficient alternatives (2, 3, 6). Microbial biodesulfurization exploits unique metabolic capabilities of certain bacteria, including *Rhodococcus* spp., to utilize fuel-borne thiophenic compounds as a sulfur source via the 4S desulfurization pathway (7–14). The 4S pathway is the most studied biodesulfurization pathway, which consists of two monooxygenases and a desulfinase encoded by a plasmid-borne operon. These enzymes catalyze the transformation of DBT to 2-hydroxybiphenyl, releasing sulfite, which can be assimilated (7, 8, 11). In addition, biodesulfurization can play an essential role in the circular economy via converting waste sulfur streams into useful products of high commercial value (6). Despite extensive research and development over several decades, low biodesulfurization catalytic activity has impeded commercial application (15–19).

Several studies suggested that physiological and metabolic traits, other than the biodesulfurization 4S pathway, can play a role in shaping the biodesulfurization activity (15, 20–22), highlighting the lack of thorough understanding of the biodesulfurization phenotype beyond the 4S pathway. While comparative systems biology studies can enable better understanding of the biodesulfurization-associated physiological and metabolic responses (23–27), such studies are very rare for desulfurizing bacteria, and most of the available studies focused on the elucidation of the desulfurization pathways without tangible insights into the underlying physiological and metabolic adaptations (28–30).

Our recent comparative proteomics and untargeted metabolomics studies on the model strain *Rhodococcus qingshengii* IGTS8 revealed unprecedented global responses in the IGTS8 strain under biodesulfurization conditions (31–33). Almost all physiological and metabolic traits underwent remodeling in terms of protein and metabolite abundance far beyond the 4S pathway. Particularly, biodesulfurizing cultures were enriched with enzymes of the novel redox cofactor mycofactocin, isocitrate lyase, C1 metabolism, and oligotrophy-related enzymes, in addition to structural rearrangements in the cell envelope. Based on the omics data, we proposed the first experimental model of sulfur assimilation in a fuel-desulfurizing bacterium and highlighted some potential targets for metabolic engineering. However, the extent of these metabolic changes and adaptations at the transcriptional level remains unclear. Integration of protein and metabolite abundance data with transcriptional regulation data can help understand the potential interaction of these differentially expressed proteins with the biodesulfurization mechanisms, which may facilitate reconstruction of alternative pathways and guide metabolic engineering strategies that can enhance the catalytic activity and efficiency of the biodesulfurization process. We performed temporal comparative transcriptional analysis of the strain *R. qingshengii* IGTS8 under biodesulfurization conditions to understand how the desulfurization-associated physiological and metabolic responses are regulated at the transcriptional level. Our findings suggest a multilevel regulation of biodesulfurization-associated adaptations, directed mainly toward cellular homeostasis and protection rather than proliferation.

## MATERIALS AND METHODS

### Culturing of *R. qingshengii* IGTS8

*R. qingshengii* IGTS8 (ATCC 53968) was grown in chemically defined medium (CDM) as described (32, 33). The culture medium contained glucose and ethanol as carbon sources and either DBT (from a 100 mM stock, dissolved in ethanol) or MgSO_4_ as a sulfur source. All cultures were in four biological replicates, and the cells were harvested at four different time points, namely, early-log phase, mid-log phase, late-log phase, and stationary phase (Fig. S1). After harvesting, the cells were washed and stored at −80°C to be used later for RNA isolation. Negative control cultures were grown in complete CDM containing glucose and ethanol as the carbon source without the addition of sulfur sources (neither DBT nor MgSO_4_). The effect of DBT on growth was tested in 100 mL CDM cultures containing 10 mM glucose and 0.1 mM of either DBT, MgSO_4_, or a mixture of both. In the MgSO_4_ cultures, an equivalent amount of ethanol (17.14 mM, the solvent of DBT) was added to exclude its effect on the growth phenotype. To verify the nitrile hydratase activity, growth on acetonitrile was tested in 100 mL CDM cultures containing acetonitrile (95.2 mM) as the sole carbon source and MgSO_4_ (0.1 mM) as a sulfur source. All cultures were incubated at 30°C in an orbital shaker (180 rpm). Growth was monitored by measuring the optical density (OD_600_).

### RNA isolation, RNA-seq library preparation, and sequencing

Total RNA was extracted from each culture using TRIzol reagent (Invitrogen) and gDNA Eliminator spin columns (Qiagen) to remove gDNA according to the manufacturer’s instructions. The RNA was dissolved in RNase-free water, quantified by NanoDrop (Thermo Fisher Scientific) and Qubit RNA BR kit (Thermo Fisher Scientific). Integrity was assessed on an Agilent 2100 Bioanalyzer (Agilent). For each sample, 117 ng of total RNA was used for library preparation with the Universal prokaryotic RNA-SEQ kit (Tecan) combined with a custom *R. qingshengii* IGTS8 rRNA AnyDeplete Probes Mix (Tecan) for ribodepletion. Sequencing was then conducted on the NextSeq2000 (Illumina) sequencing platform with NextSeq 1000/2000 P2 Reagents V3 (Illumina) and SR 1 × 100 cycles sequencing mode.

### Differential analysis of the transcriptomes

Transcriptomic reads were trimmed by Trimmomatic v0.38.0 using a quality threshold of 20 across a sliding window of 4 bases, and transcripts were quantified by mapping them to coding sequences of *R. qingshengii* IGTS8 using Kallisto quant (34). An expression matrix was generated from the abundance estimates using Trinity (35). Differential expression of normalized-trimmed mean of M-values was analyzed using DESeq2 between different culture conditions (36). All analyses were performed using the Galaxy web-based platform (37). The changes in transcript abundance relative to the control group (MgSO_4_ cultures) were expressed as log_2_ fold change, and the genes with log_2_ fold change ≥ 2 or ≤ −2 and *P* ≤ 0.05 were regarded as differentially expressed genes (DEGs).

### Real-time quantitative PCR

To validate the RNA-seq results, the relative expression of several randomly selected genes was assessed by qPCR. In total, 16 genes were selected to represent different relative abundance categories according to the RNA-seq profile (Table S1). Briefly, 350 ng of each RNA sample was used to perform first-strand cDNA synthesis using the LunaScript RT SuperMix Kit with oligo-dT and random primers (New England Biolabs) according to the manufacturer’s instructions. The primers were intron-spanning, designed using LightCycler Probe Design 2 software (Roche). Real-time quantitative PCR (RT-qPCR) was performed using the Takyon No ROX SYBR 2X MasterMix blue dTTP (Eurogentec) according to the manufacturer’s instructions on an LC480-II real-time PCR system (Roche). For each sample, technical and biological replicates were included. Melting curve analysis was performed for each gene to confirm the primers’ specificities. Log_2_ fold changes in relative abundance of target transcripts were calculated by the ΔΔCt method with primers efficiencies correction by LinReg software and normalization with IGTS8_peg2303 and IGTS8_peg1284 reference genes (Table S1).

### Functional classification of the differentially expressed genes

Functional inferences for the gene products were based on similarity searches performed using the BLAST program at the NCBI and UniProt databases with default settings. Mapping of the proteins to metabolic pathways was performed with the KEGG Mapper by BlastKOALA (https://www.kegg.jp/blastkoala/). To perform functional classification of the DEGs, a BLAST fasta 36 search was performed (38) against a public *Rhodococcus erythropolis* genome from UniProtKB/Swiss-Prot. For each gene, Gene Ontology (GO) terms were queried from the Gene Ontology database (http://geneontology.org/), and proteins were filtered according to their functional classification.

### Enrichment analysis of the differentially expressed genes

Functional annotation enrichment analysis of differential transcriptomic data was performed for each growth phase in an unbiased fashion using the desktop version of DAVID (Ease v2.0) (39, 40) and an updated version of GO and KEGG pathway databases (18 October 2021). The list of DEGs from each comparison (DBT and MgSO_4_ cultures) was compared to the list of all matches identified. Enriched GO terms were filtered by only considering those with an Ease score lower than 0.1 and a Benjamini *P* value lower than 0.05.

## RESULTS

### Overview of the transcriptomes

**Fig 1:**
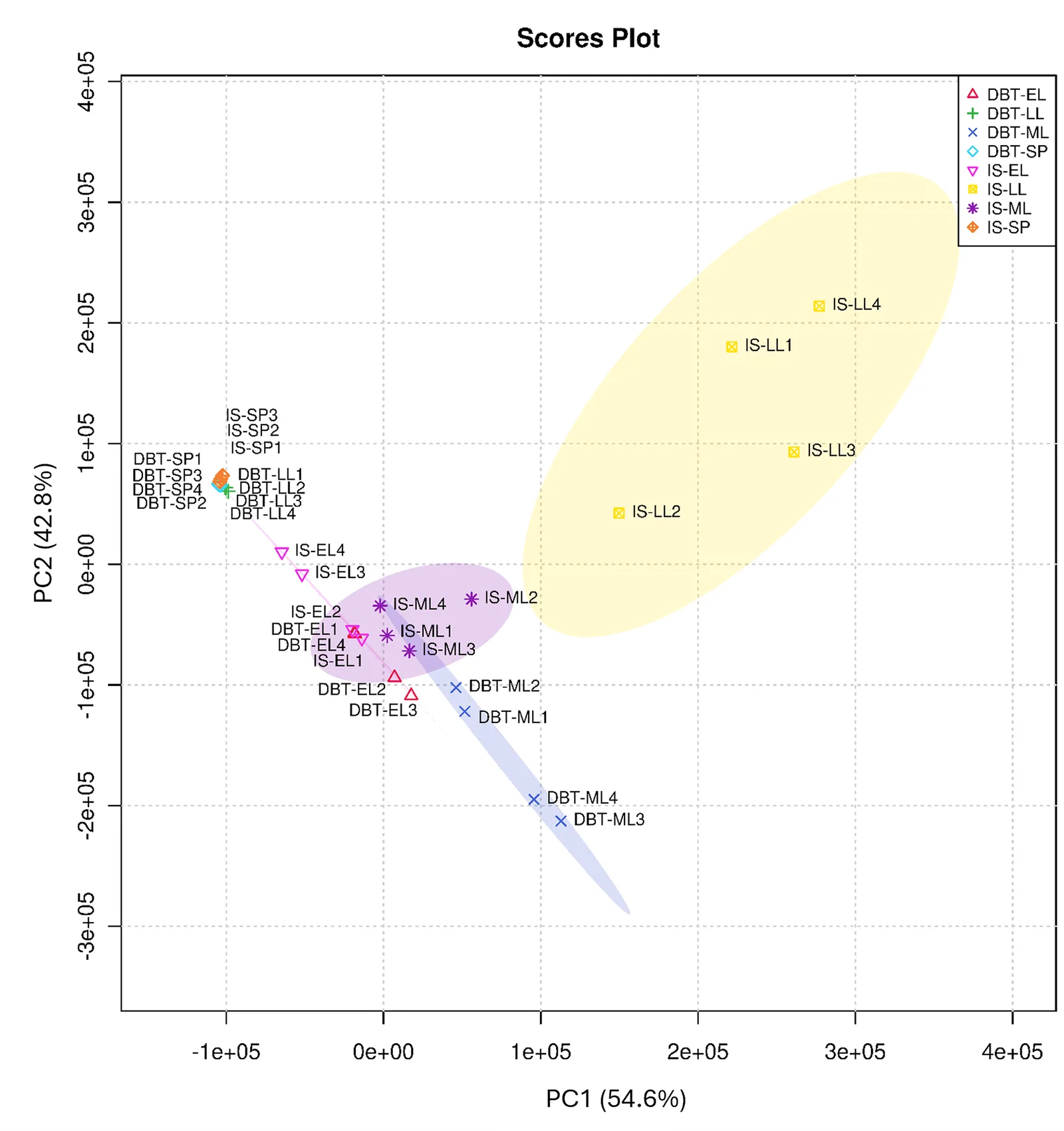
Principal component analysis of the DEGs using the two main dimensions. DBT indicates the dibenzothiophene culture and IS indicates the inorganic (MgSO_4_) culture. The growth phases are abbreviated as follows: EL (early-log), ML (mid-log), LL (late-log), and SP (stationary phase). The numbers shown after the growth phase indicate the number of the replicates.

The fuel-biodesulfurizing strain *R. qingshengii* IGTS8 grew in CDM and utilized either DBT or MgSO_4_ as the sulfur source (Fig. S1 and S2). Of the 6,734 protein-coding genes of *R. qingshengii* IGTS8, we detected 6,690 transcripts (coverage of 99.5%). The relative abundance of many transcripts varied significantly depending on the type of the sulfur source and growth phase (Table S2, Fig. S3; Fig. 1). Many of the DEGs encode hypothetical proteins and ABC-type transporters. In the DBT culture, 623 DEGs were upregulated and 587 DEGs were downregulated at least during one growth phase.

**Fig 2:**
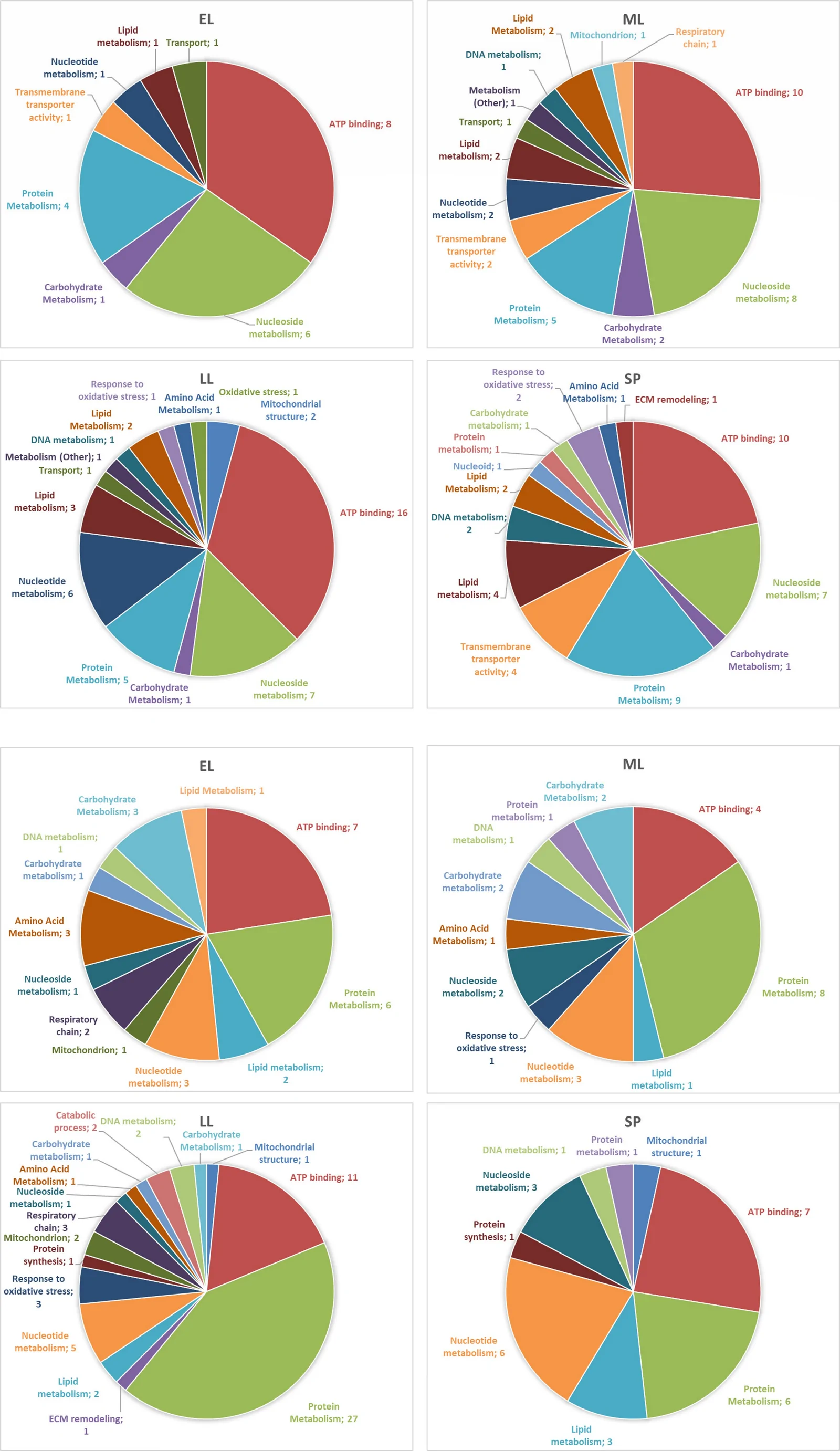
Functional classification of the DEGs in *R. qingshengii* IGTS8. The upper four panels show genes upregulated in the DBT culture, and the lower four panels show genes downregulated in the DBT culture. The growth phases are abbreviated as follows: EL (early-log), ML (mid-log), LL (late-log), and SP (stationary phase). The pie charts indicate the numbers of DEGs identified in each functional category. The numbers of DEGs are shown next to each category.

Among the detected transcripts, only 1,399 could be assigned to functional categories according to GO terms from the Gene Ontology database (Fig. 2). Most transcripts belonged to the major metabolic and physiological processes, such as metabolism of carbohydrates, lipids, proteins, nucleotides, amino acids, stress response, transcription, etc. However, the majority of differential expression was observed among genes involved in ATP-binding, nucleoside/nucleotide metabolism, and protein metabolism.

Enrichment analysis revealed that no GO terms were significantly enriched during the early-log phase (Fig. S4). The highest number of enriched genes were observed in the late-log phase cultures. These encoded structural constituents of ribosomes or were involved in cellular metabolic processes and other molecular activities. In contrast, the stationary phase cells had the lowest number of enriched gene categories, which are involved mainly in inorganic ion transport.

**Fig 3:**
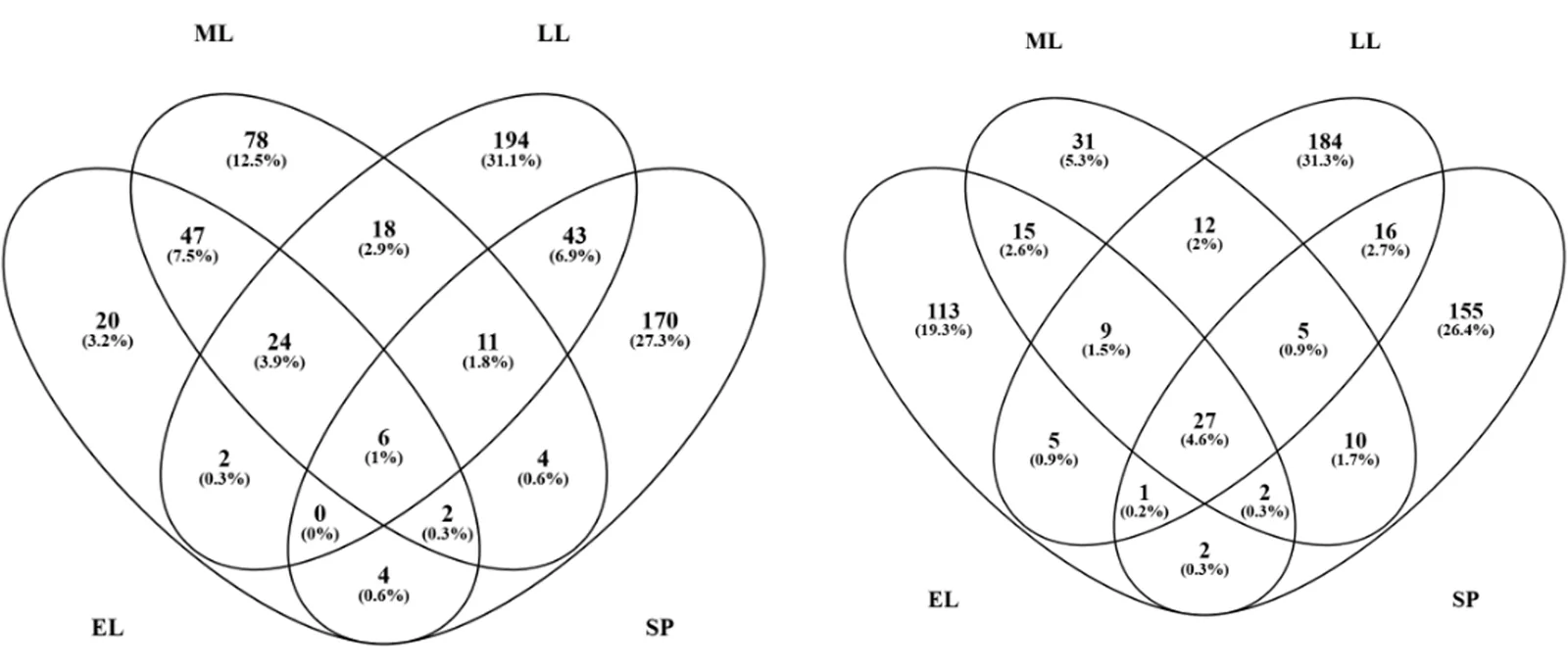
Venn diagram of the DEGs in the four growth phases for the upregulated (left) and downregulated (right) genes in DBT culture using Venny tool (v 2.1). The growth phases are abbreviated as follows: EL (early-log), ML (mid-log), LL (late-log), and SP (stationary phase).

Only 1% of the significantly overexpressed genes in the DBT culture were commonly found in the four growth phases (Fig. 3). Most of those genes encode hypothetical proteins. However, 4.6% of the significantly downregulated genes were shared by cells from the four growth phases, with genes encoding hypothetical proteins and proteins of inositol phosphate metabolism being the most common (Tables S3 and S4). In both cases, cultures of the late-log phase had the highest number of exclusively detected genes, thus aligning well with the PCA results, which show that the strongest transcriptional response of the IGTS8 occurred during the late-log phase. To validate the RNA-seq results, the expression of several genes was investigated by qPCR. The overall expression profile of the selected genes was consistent with the RNA-seq profile, with minor differences in the magnitude of expression (Table S5) as reported in similar studies (41).

### Sulfur metabolism under biodesulfurization conditions

**Fig 4:**
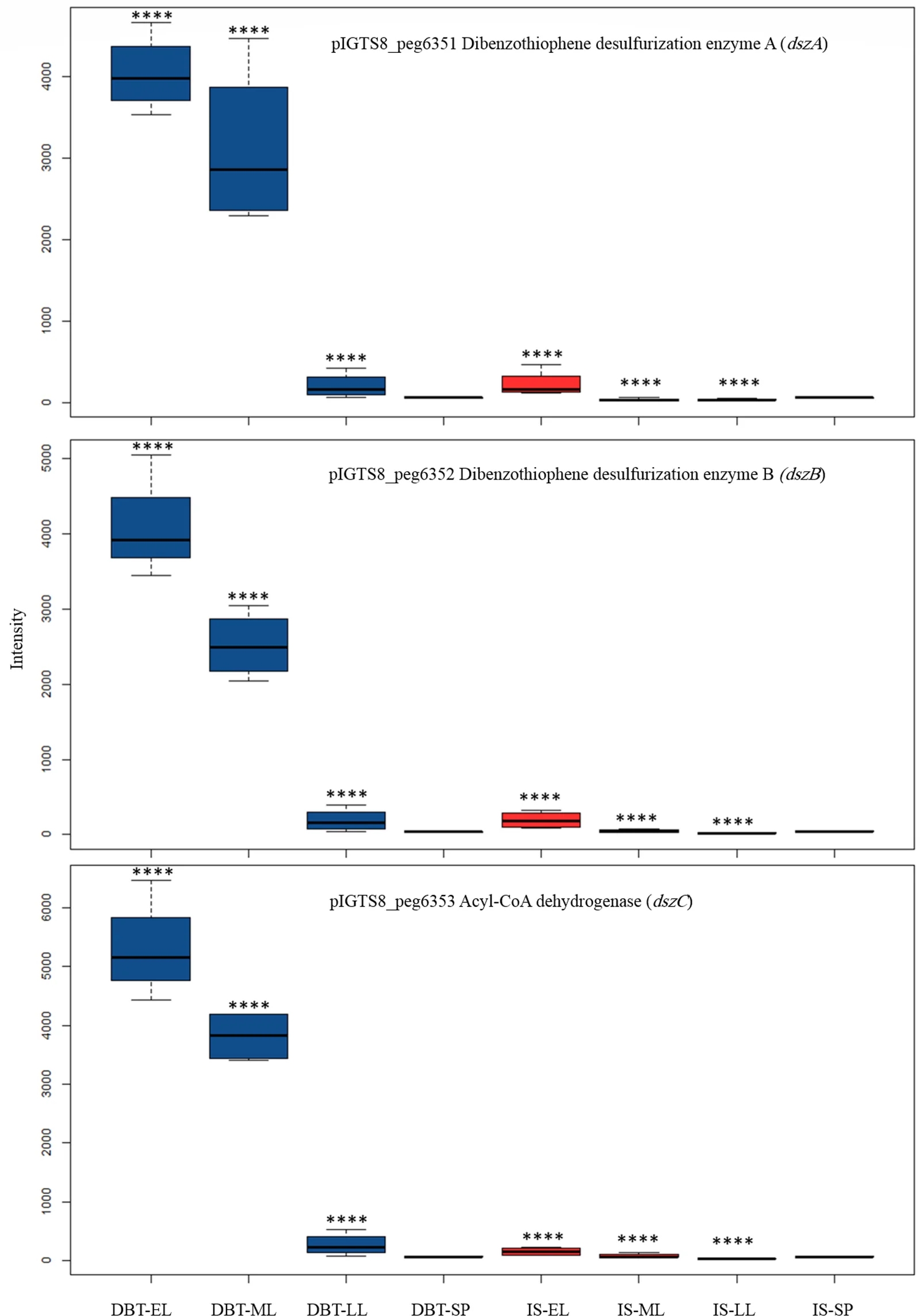
Boxplots representing relative gene expression values showing the abundance profile of the transcripts of the 4S desulfurization pathway genes in the dibenzothiophene (DBT, in blue) and inorganic sulfate (IS, in red) cultures. The growth phases are abbreviated as follows: EL (early-log), ML (mid-log), LL (late-log), and SP (stationary phase). The relative gene expression in the DBT culture was compared to that in the MgSO_4_ culture. Significance is indicated as follows: no symbol, *P* > 0.05; *, *P* ≤ 0.05; **, *P* ≤ 0.01; ***, *P* ≤ 0.001; ****, *P* ≤ 0.0001. The boxplots were drawn in the software R (4.0) (32, 33).

There were significant alterations in the expression of many genes involved in sulfur source uptake, sulfur acquisition and assimilation, and biosynthesis of key sulfur-containing metabolites (Tables S2 and S6). The 4S desulfurization pathway genes (pIGTS8_peg6351-pIGTS8_peg6353) were consistently and significantly upregulated in the DBT culture starting from the early-log phase (log_2_ fold change = 2.34 to 6.5) (Fig. 4). This expression profile is consistent with the reported higher content of the 4S pathway enzymes and intermediates (dibenzothiophene sulfoxide and dibenzothiophene sulfone) in the DBT culture (32). However, the relative abundance of these genes in the DBT culture dropped significantly during the stationary phase, potentially due to lower desulfurization needs. Several genes encoding ABC-type transporters, sulfatases, and oxygenases involved in the uptake and utilization of alternative sulfur sources were overexpressed in the DBT culture, except during the stationary phase. Genes encoding sulfate activation and reduction are clustered in an operon (IGTS8_peg2383-2387), and their expression did not vary significantly between the two cultures. However, downregulation of these genes was observed in the DBT culture during the stationary phase.

**Fig 5:**
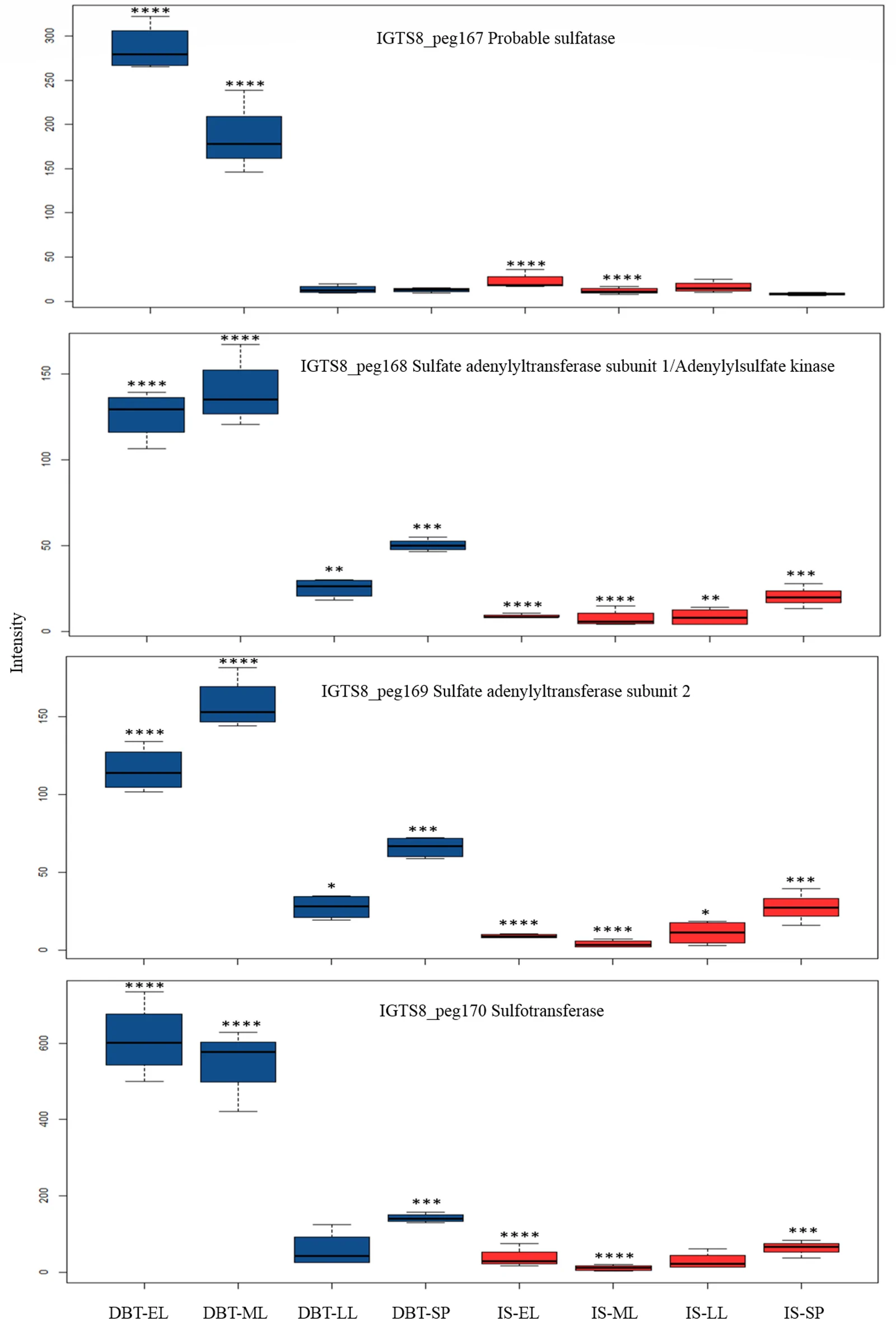
Boxplots representing relative gene expression values showing the abundance profile of the transcripts of some sulfur metabolism genes in the dibenzothiophene (DBT, in blue) and inorganic sulfate (IS, in red) cultures. The growth phases are abbreviated as follows: EL (early-log), ML (mid-log), LL (late-log), and SP (stationary phase). The relative gene expression in the DBT culture was compared to that in the MgSO_4_ culture. Significance is attested as follows: no symbol, *P* > 0.05; *, *P* ≤ 0.05; **, *P* ≤ 0.01; ***, *P* ≤ 0.001; ****, *P* ≤ 0.0001. The boxplots were drawn in the software R (4.0) (32, 33).

Sulfite released from DBT via the 4S pathway could be directly reduced to sulfide by CysI (ferredoxin sulfite reductase), which was significantly downregulated in the DBT culture during the stationary phase. A gene encoding a putative sulfite oxidase/oxidoreductase (IGTS8_peg2618) was overexpressed in the DBT culture during the mid-log phase (log_2_ fold change = 2.5). Moreover, a putative YeiH protein, which is 28% identical to a YeiH family sulfate exporter and is encoded by the IGTS8_peg446 gene, was downregulated under biodesulfurization conditions. Interestingly, transcripts of a bifunctional sulfate adenylyltransferase/APS kinase sulfate activation complex (CysN2CD2) were significantly more abundant in the DBT culture during the early-log and mid-log phases. Downstream of CysD2, IGTS8_peg170 encodes a sulfotransferase (Stf0) and was similarly overexpressed during the early-log and mid-log phases (Fig. 5). Among the genes of methionine biosynthesis, *metY* (IGTS8_peg3888) was significantly upregulated in the DBT culture except during the stationary phase. The expression of *metE* (IGTS8_peg5907) was significantly higher only during the mid-log phase. Consistent with this finding, metabolomics analyses showed that the methyl group donor for MetE (5-methyltetrahydropteroyltri-L-glutamate) was more abundant in the dibenzothiophene culture during the early-log phase and decreased afterward (32).

The expression of several genes encoding predicted nitrilotriacetate (NTA) monooxygenases (IGTS8_peg3535, IGTS8_peg5732, IGTS8_peg3467) increased and was higher in the DBT culture, particularly during the early- and mid-log phases. NTA monooxygenases catalyze the transformation of NTA to iminodiacetate and glyoxylate. They belong to a subclass of flavoprotein monooxygenases which includes alkanesulfonate monooxygenase (SsuD) (42, 43). The protein encoded by the IGTS8_peg5732 gene is 48.6% identical (E-value: 2.6e-148) to N-acetyl-S-(2-succino)-L-cysteine monooxygenase from *Bacillus subtilis* strain 168 (UniProt KB-P54950, SCMK-BACSU) (34). This enzyme catalyzes the oxygenation of the succinyl moiety of N-acetyl-S-(2-succino)-L-cysteine, which generates oxaloacetate and N-acetylcysteine. This constitutes a part of the S-(2-succino)-L-cysteine degradation pathway, allowing *B. subtilis* to grow on it as a sole sulfur source via cysteine. Metabolism of S-(2-succinyl)-L-cysteine produces cysteine, thus making this enzyme a part of cysteine biosynthesis (44).

### Biodesulfurization-associated carbon metabolism

**Fig 6:**
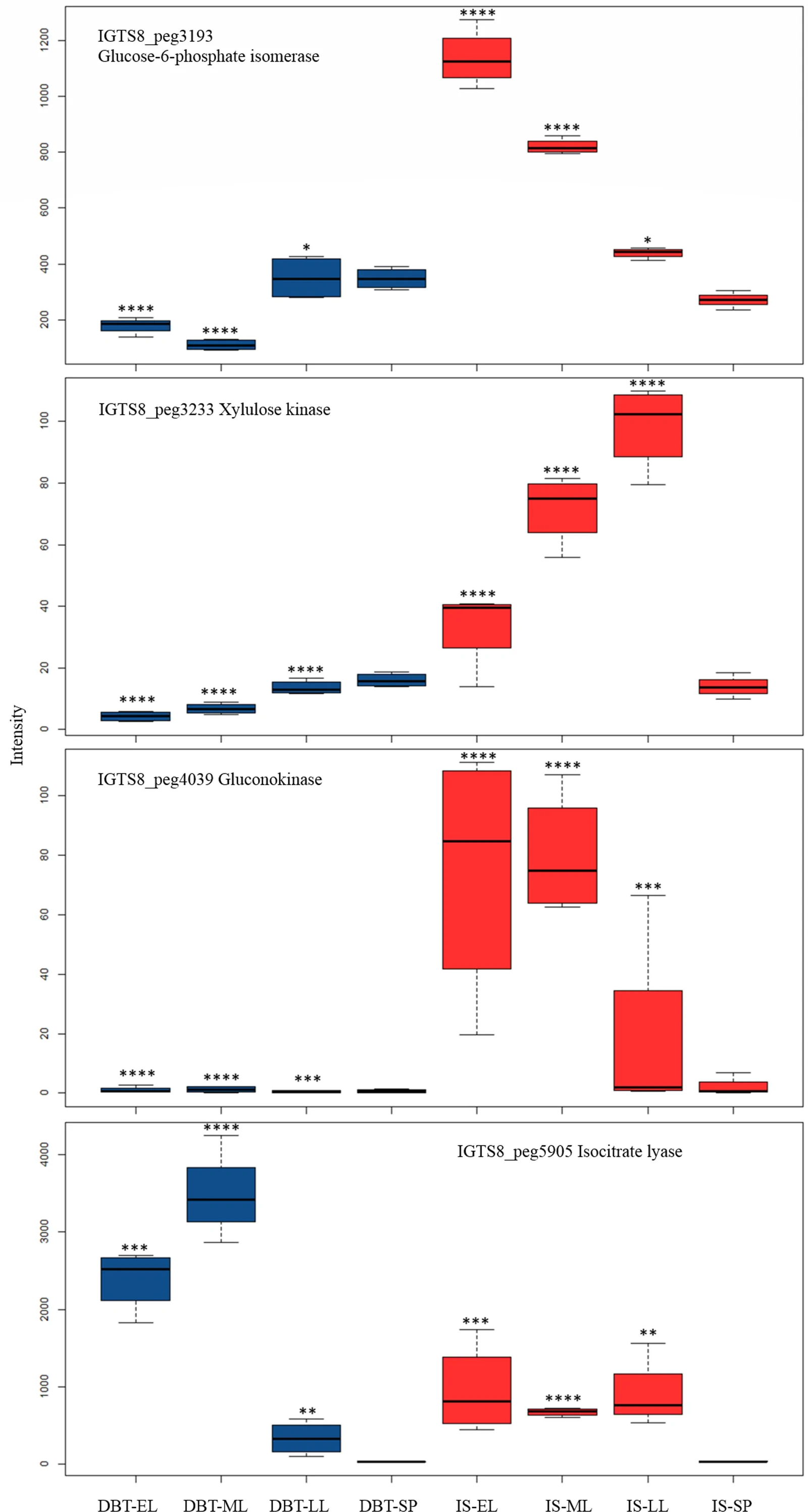
Boxplots representing relative gene expression values showing the abundance profile of the transcripts of carbohydrate and central metabolism genes in the dibenzothiophene (DBT, in blue) and inorganic sulfate (IS, in red) cultures. The growth phases are abbreviated as follows: EL (early-log), ML (mid-log), LL (late-log), and SP (stationary phase). The relative gene expression in the DBT culture was compared to that in the MgSO_4_ culture. Significance is attested as follows: no symbol, *P* > 0.05; *, *P* ≤ 0.05; **, *P* ≤ 0.01; ***, *P* ≤ 0.001; ****, *P* ≤ 0.0001. The boxplots were drawn in the software R (4.0) (32, 33).

Most of the genes associated with carbohydrate and central metabolism were either downregulated in the DBT culture or exhibited no significant difference in expression levels (Table S2; Fig. 6). Consistent with these findings, our recent metabolomics studies revealed lower abundance of some carbohydrate and central metabolites under biodesulfurization conditions, including xylulose-5-phosphate/ribose-5-phosphate, fructose-6-phosphate, fumarate, glucose-6-phosphate, and succinate (33). Exceptionally, the gene encoding isocitrate lyase (IGTS8_peg5905), a key enzyme of the glyoxylate shunt, was significantly upregulated in the DBT culture (log_2_ fold change = 2.4) during the mid-log phase (Fig. 6). Moreover, a gene cluster predicted to encode enzymes for inositol phosphate metabolism (IGTS8_peg782-789) and a related GentR family transcriptional regulator were significantly downregulated under biodesulfurization conditions during all growth phases (Tables S2 and S4) (33). This gene cluster includes some of the most significantly downregulated genes detected in the RNA-seq data (log_2_ fold change ~ −5.0 up to −9.0). KEGG pathway analysis mapped many of those genes in the inositol phosphate metabolism pathway under the category carbohydrate metabolism (Fig. S5). Upstream, three clustered genes annotated as inositol transport system sugar-binding protein (IGTS8_peg800), inositol transport system permease protein (IGTS8_peg801), and inositol transport system ATP-binding protein (IGTS8_peg802) were also downregulated (log_2_ fold change ~ −2.5 up to −7.0) in the DBT culture during all growth phases (Tables S2 and S6). KEGG mapper analysis showed that the gene products constitute a simple sugar transport system (K02056, K02057, K02058). Consistent with their role in sugar transport, a UniProt BLAST search revealed that IGTS8_peg801 gene product is identical to the xylose transport system permease protein (XylH) from *Rhodococcus baikonurensis* (A0ABV5X8Z9). The close location to inositol phosphate metabolism genes suggests that these genes potentially constitute a putative inositol transporter.

In addition, five genes annotated to encode carbonic anhydrase were found in the IGTS8 genome. One of these (IGTS8-peg997) was significantly upregulated in the DBT culture during the early-log and mid-log phases. Another gene (IGTS8-peg5020) was significantly upregulated in the DBT culture during the late-log phase, and its expression significantly decreased during the stationary phase. The expression of the other three predicted carbonic anhydrase genes was not significantly different between the DBT and MgSO_4_ cultures. It is, however, worth noting that we could not detect any carbonic anhydrases in our recent proteomics investigation on the IGTS8 strain (33). One possibility could be their location in the cell membrane. To find out whether this is the case, we analyzed the protein sequence of the five predicted carbonic anhydrases in the IGTS8 genome using a transmembrane region prediction tool (31) (https://services.healthtech.dtu.dk/services/DeepTMHMM-1.0/). Among the five carbonic anhydrases encoded in the IGTS8 genome, only one (IGTS8_peg3341) was predicted to be membrane-associated with 12 transmembrane regions (Fig. S6).

Three genes (IGTS8_peg6110-6112) clustered in an operon and encoding MoxR/CoxE/ThcE orthologs are probably involved in oligotrophy or C1 metabolism, as shown recently (33). These genes were upregulated in the DBT culture, albeit a significant difference was noticed only during the stationary phase. Transcripts of the operon (IGTS8_peg4820-4823) were detected in the RNA-seq data in both the DBT and MgSO_4_ cultures, mostly with no significant differences in the relative abundance. Only one of them (IGTS8_peg4820) was significantly upregulated in the DBT culture during the late-log phase (log_2_ fold change = 2.3). These genes are predicted to code for the structural subunits of a putative CO dehydrogenase (33).

### Amino acid degradation is stimulated in the DBT culture

Genes encoding the large and small chains of glutamate synthase were differentially expressed in the DBT culture. The large chain gene was strongly downregulated during the mid-log phase, whereas the small chain transcript was significantly more abundant during the late-log phase. Moreover, many genes of valine/leucine/isoleucine and proline degradation were significantly upregulated in the DBT culture (Table S2). Sarcosine oxidase transcripts were enriched in the DBT culture during the early-log and mid-log phases (log_2_ fold change = 3.9). One of the glycine cleavage system genes (IGTS8_peg1817, encoding the P protein) was overexpressed in the DBT culture during the late-log phase. The protein encoded by gene IGTS8_peg1817 is 80.4% identical to glycine dehydrogenase (GcvP, decarboxylating) from *Rhodococcus fascians* (Q8G9M2, UniProtKB reviewed). Interestingly, genes of aromatic amino acid biosynthesis were also differentially expressed depending on the sulfur source and growth phase. The gene IGTS8_peg2445, encoding quinate/shikimate 5-dehydrogenase, was downregulated, whereas 3-dehydroquinate dehydratase II transcript was more abundant in the DBT culture. Glutamate dehydrogenase (NADP-specific) gene was significantly underexpressed in the DBT culture during the late-log phase. A gene cluster encoding valine degradation enzymes was overexpressed in the DBT culture during the stationary phase (Table S2). In addition, biodesulfurization stimulated a higher expression level of genes encoding enzymes of proline degradation to glutamate. A gene cluster encoding different urease subunits and accessory proteins (IGTS8_peg342-348) was consistently upregulated in the DBT culture during the late-log phase after an initial expression downshift during the early-log phase. The DBT culture had a significantly lower abundance of the transcripts of a urea ABC transporter encoded by an operon (IGTS8_peg5886- IGTS8_peg5890) starting from the early-log phase (log_2_ fold change = −2.34 to −3.8).

Biodesulfurization stimulated the expression of genes encoding the alpha and beta subunits of a nitrile hydratase (IGTS8_peg4868, IGTS8_peg4869), which were significantly upregulated (log_2_ fold change up to 6.76) in the DBT culture during the late-log and stationary phases (Table S2). Upstream to these genes, a gene encoding an amidase (IGTS8_peg4867) was significantly upregulated in the DBT culture during the mid-log and late-log phases (log_2_ fold change = 2.8 to 4.4). A putative metal chaperone, probably involved in nitrile hydratase activation, is encoded downstream of the nitrile hydratase genes (IGTS8_peg4870) and was also significantly upregulated during the late-log phase. The protein sequence of this putative activator contains the conserved cysteine-rich motif (C-X-C-C) common to all Fe-type activators of nitrile hydratases (45). This gene order aligns with the organization of the operons encoding Fe-type nitrile hydratases (46). Nitrile hydratases are key biocatalysts for the industrial conversion of nitriles into amides. Moreover, they can be applied in bioremediation, where they degrade toxic nitriles, such as acetonitrile, adiponitrile, and acetamiprid (46). In addition to detoxification of intracellular nitriles, nitrile hydratases play a role in nitrogen metabolism by releasing ammonia, which can be assimilated. It is also known that these enzymes are involved in stress adaptations, where they are induced under oxidative or nutrient stress. The metal ion in the enzyme active site contributes to redox regulation and protection from oxidative damage (46–48). The activity of nitrile hydratases in the strain IGTS8 was confirmed by its ability to grow in CDM containing acetonitrile as the sole carbon source (Fig. S7).

### Remodeling of lipids and fatty acids metabolism

Transcripts of many lipid and fatty acid metabolism genes were detected, including those related to fatty acid biosynthesis and degradation, glycerophospholipid metabolism, as well as triacylglycerol and diacylglycerol metabolism (Table S2). The relative abundance of these transcripts was not significantly different in most cases, with some exceptions. The gene encoding a putative malonyl-CoA acyl carrier protein transacetylase (IGTS8_peg459), involved in fatty acid and polyketide biosynthesis, exhibited a lower expression (log_2_ fold change of −3.8) in the DBT culture during the stationary phase. On the contrary, transcripts of the acyl carrier protein gene were significantly more abundant in the DBT culture during the mid-log and stationary phases. Significant upregulation of the gene encoding long-chain fatty acid-CoA ligase (Mycobacteria subgroup) occurred during the mid-log (log_2_ fold change = 4.5) and late-log phase (log_2_ fold change = 2.4). The expression levels of several fatty acid metabolism genes increased toward the stationary phase in the DBT culture after initial downshift. Two 3-oxoacyl-(acyl carrier protein) reductase genes were also downregulated in the DBT culture during the stationary phase. On the contrary, there was an upshift (log_2_ fold change = 3.06) in the abundance of the transcript of acetoacetyl-CoA synthase during the stationary phase under biodesulfurization conditions. Biodesulfurization also stimulated the expression of four clustered genes during the late-log phase, which are predicted to encode malonyl-CoA acyl carrier protein transacylase (IGTS8_peg2304), acyl carrier protein (IGTS8_peg2305), 3-oxoacyl-[acyl-carrier-protein] synthase KASII (IGTS8_peg2306), and propionyl-CoA carboxylase beta chain (IGTS8_peg2307). The IGTS8_peg2305 gene product is 98.95% identical to acyl carrier protein from *R. erythropolis* PR4/NBRC 100887 (C1A96, UniProtKB reviewed). In addition, the IGTS8_peg2307 gene product is 95.4% identical to propionyl-CoA carboxylase beta chain from *R. erythropolis* (Q06101, UniProtKB).

### Transcriptional changes in the mycofactocin system

Some of the mycofactocin biosynthesis genes were differentially expressed based on the type of the sulfur source and growth phase (Table S2). For instance, transcripts of the mycofactocin system small protein, mycofactocin radical SAM maturase, mycofactocin system heme/flavin dehydrogenase, and mycofactocin system transcriptional regulator were significantly more abundant in the DBT culture during some growth phases. Moreover, the mycofactocin precursor gene was significantly downregulated in the DBT culture in the late-log phase before it shifted upward during the stationary phase. Many of the mycofactocin biosynthesis and associated proteins were detected with much higher abundance in the DBT culture according to our recent comparative proteomics studies (33). Mycofactocin is a glycosylated redox cofactor that is ribosomally synthesized and post-translationally modified. It is commonly found in *Mycobacterium* spp. and other *Actinomycetota*, including rhodococci (49). It was proposed to play roles in survival under hypoxia, redox homeostasis, oligotrophy, as well as in glucose and alcohol metabolism (49–51).

### Changes in the cell envelope and cell division alterations in the DBT culture

Consistent with the recently reported proteomics and metabolomics studies (33), over 50 genes involved in cell envelope biosynthesis and degradation were differentially expressed in DBT culture, including genes encoding transglycosylases, transpeptidases, endopeptidases, murein hydrolases, peptidoglycan recycling, as well as cell surface-associated proteins (Table S2). Several cell division and peptidoglycan biosynthesis genes were significantly upregulated in the DBT culture during the stationary phase, with the exception of the gene encoding D-alanyl-D-alanine carboxypeptidase. However, in most cases, the differences in transcript abundance were not significant. The most conspicuous differences were noted for the expression of an operon (IGTS8_peg5608-5610) encoding enzymes of fucose (O-antigen nucleotide sugar) biosynthesis. The three genes were significantly overexpressed under biodesulfurization conditions, and KEGG pathway analysis revealed that they are involved in the biosynthesis of nucleotide sugars (KEGG Pathway: map00541). The abundance of the N-acetylmuramoyl-L-alanine amidase gene transcript was significantly higher in the DBT culture only during the stationary phase (log_2_ fold change = 3.4). This expression profile was also noted for genes encoding the cell division proteins FtsI (IGTS8_peg2138), FtsL (IGTS8_peg2139), and MraZ (IGTS8_peg2141). MraZ is a transcriptional regulator that negatively regulates its own expression and the expression of cell division and cell wall-related genes (the cell wall cluster). The IGTS8_peg2141 gene product is 99.3% identical to the transcriptional regulator MraZ from *Rhodococcus erythropolis* (strain PR4/NBRC 100887) (C1A0Y4, UniProtKB reviewed). The PR4 protein acts as a negative regulator of DNA-templated transcription initiation. This may explain why the *mraZ* gene was overexpressed in the DBT culture during the stationary phase. When overproduced, MraZ is lethal and perturbs cell division. However, the cells can be protected from the deleterious impact of MraZ overproduction by co-overproduction of the rRNA small subunit methyltransferase (RsmH) (52). The expression of a gene encoding RsmH (IGTS8_peg2140), downstream of *mraZ*, increased in the DBT culture during the stationary phase and was significantly more abundant (log_2_ fold change = 3.96) (Tables S2 and S6). Analysis of the IGTS8_peg2140 protein sequence revealed 99.4% identity to ribosomal RNA small subunit methyltransferase H from *Rhodococcus erythropolis* strain PR4/NBRC 100887 (UniProt accession number: C1A0Y3). This enzyme specifically methylates the N4 position of cytidine at position 1402 (C1402) of 1S rRNA. Several glycosyltransferase genes associated with exopolysaccharides, capsular polysaccharides, O-antigen, and teichoic acid export were overexpressed in the biodesulfurizing culture during the late-log and stationary phases. In addition, genes predicted to encode chromosome partitioning and septum formation proteins were significantly downregulated in the DBT culture during the early-log phase (Tables S2 and S6).

### General stress response was activated during DBT desulfurization

Under biodesulfurization conditions, diverse stress-related genes exhibited differential expression, which was also dependent on the culture age. The expression profile of those genes was not consistent and varied between upshift and downshift in the transcript levels compared to MgSO_4_-grown cultures (Table S2). Most of the differentially expressed genes in this category encode oxidative stress proteins, chaperones, universal stress proteins, peptide transport systems, stress-related sigma and anti-sigma factors, as well as transcriptional regulators of cell division and nutrient starvation. The universal stress protein-encoding gene was significantly upregulated in the DBT culture starting from the late-log phase. On the contrary, a gene encoding a putative Asp23/Gls24 family envelope stress response protein (IGTS8_peg5580) was downregulated in the late-log (log_2_ fold change = −2.6) and stationary phases (log_2_ fold change = −3.66). This protein is similar to *Staphylococcus aureus* alkaline shock protein 23 (Asp23) and *Streptococcus pyogenes* stress response regulator Gls24 homolog (53, 54). The IGTS8_peg5580 gene is also identical to an Asp23/Gls24 family envelope stress response protein from *R*. *baikonurensis* (UniProt accession number: A0ABV5XBT0) (Tables S2 and S6). Three clustered genes (IGTS8_peg2423-2425) encoding ectoine biosynthesis enzymes exhibited differential expression in the DBT culture. Two of the enzymes encoded by this gene cluster were detected in higher abundance under biodesulfurization conditions in our recent proteomics and metabolomics studies, consistent with a higher level of ectoine (33).

Moreover, the transcript levels of the gene IGTS8_peg5912 were significantly more abundant under biodesulfurization conditions (log_2_ fold change up to 2.8). This gene encodes a STAS (Sulfate Transport and Anti-Sigma factor antagonist) domain-containing protein similar to *Streptomyces coelicolor* anti-sigma-B factor antagonist (Tables S2 and S6). Anti-anti-sigma factors play an important role in the regulation of several sigma factors and their corresponding anti-sigma factors. The STAS domain is also found in the C-terminal region of sulfate transporters and stressosomes, suggesting a role under sulfate starvation (55). In addition, several putative ABC-type dipeptide/oligopeptide transport gene transcripts were more abundant in the DBT culture, particularly during the stationary phase. Many of those genes are clustered in operons, such as IGTS8_peg3720-3724 and IGTS8_peg5788-5791 (31, 33). Biodesulfurization was also associated with higher expression of a gene encoding cold shock protein CspA (IGTS8_peg4431) during late-log (log_2_ fold change = 4.62) and stationary phase (log_2_ fold change = 4.23). UniProt analysis revealed similarity of the IGTS8_peg4431 protein sequence to cold shock proteins from different bacteria. For instance, it is 59.7% identical (E-value: 2.1e-19) to a cold shock protein from *Streptomyces dubilierae* (A0ABU2PIR4) (Tables S2 and S6). These findings were further backed by KEGG mapper analysis, which classified IGTS8_peg4431 as a transcription factor related to cold shock (Orthology: K03704) (56).

Genes encoding toxin-antitoxin systems, such as programmed cell death toxin *ydcE* (pIGTS8_peg6682) and “prevent host death” protein (Phd antitoxin) (pIGTS8_peg6638), were significantly upregulated in the DBT culture during the late-log and mid-log phases, respectively (Tables S2 and S6). Conserved domain analysis classified the pIGTS8_peg6682 gene product as the type II toxin-antitoxin system PemK/MazF family toxin (ArchID 10006385). It is similar to *Staphylococcus aureus* MazF, a ribosome-independent sequence-specific endoribonuclease that cleaves at 5′-UACAU-3′ sequences of RNA and is the toxin component of the MazF-MazE TA module (57, 58). The protein encoded by pIGTS8_peg6682 is 99.2% identical (E-value: 3.5e-76) to an mRNA interferase from *R. erythropolis* (UniProt accession number: A0A1f2PUJ5), a toxin component of a type II toxin-antitoxin system. It also exhibits 59.8% identity (E-value: 2.2e-38) to endoribonuclease MazF9 from *Mycobacterium tuberculosis* (strain ATCC 25618/H37Rv) (UniProt accession number: P71650). This transcriptional response reflects the role of bacterial toxin-antitoxin systems in adaptation to several stressors, including oxidative stress and nutrient starvation (58, 59).

The expression of some genes encoding iron transport proteins generally exhibited a downshift in the DBT culture, probably to mitigate oxidative stress (Tables S2 and S6). Among them, the gene cluster IGTS8_peg4619-4620 encodes a component of a putative iron chelate ABC-type transport system. According to a BLAST search in UniProt, the protein sequences of IGTS8_peg4621 and IGTS8_peg4622 are 99.7% identical (E-value: 0.0) to iron chelate uptake ABC transporter family permease subunits from *R. qingshengii* JCM 15477 (A0AB38RDC8, A0AB38RBH5). The substrate-binding subunit is encoded by the gene IGTS8_peg4623, whose protein product is identical to Fe^2+^-enterobactin ABC transporter substrate-binding protein (FepB) from *R. qingshengii* JCM 15477 (A0AB38RCF3). The remaining two genes of this gene cluster encode proteins with high identity to siderophore-interacting proteins, ATP-binding proteins of ABC-type transporters, and SIP domain-containing proteins.

Moreover, the DBT culture had a lower abundance of transcripts related to tRNA modification, such as tRNA (Guanine 37-N1)-methyl transferase (IGTS8_peg1146) and pseudouridine synthase (IGTS8_peg642), particularly during the late-log phase. KEGG mapper analysis showed that both IGTS8_peg642 and IGTS8_peg1146 are classified under the ortholog of tRNA biogenesis (ko03016). In agreement with the KEGG mapper findings, IGTS8_peg642 showed 99.7% identity to tRNA pseudouridine synthase A (TruA) from *R. qingshengii* JCM 15477 (UniProtKB, A0AB38RIC0). In addition, IGTS8_peg1146 is identical (E-value: 2.9e-170) to tRNA (guanine-N(1)-)-methyltransferase (TrmD) from *R. qingshengii* JCM 15477 (UniProtKB, A0AB38RKD9) (Tables S2 and S6).

Biodesulfurization increased the expression of the IGTS8_peg5867 gene, which encodes a polyphosphate kinase during the late-log and stationary phases. Conserved domain analysis showed that it is a polyphosphate kinase 2 (PPK2) family protein similar to bacterial PPK2, which catalyzes polyphosphate-dependent phosphorylation of nucleoside diphosphates (ADP, GDP) to nucleoside triphosphates (60). The IGTS8_peg5867 gene product was classified under the categories energy metabolism (00190) and RNA degradation (03018) according to KEGG mapper analysis.

While genes related to iron, potassium, and zinc transport were downregulated in the biodesulfurizing culture, phosphate and manganese transport genes were overexpressed (Table S2). Genomic analysis revealed that most of those genes are clustered in putative operons. For instance, the gene cluster IGTS8_peg151-154 encodes subunits of a putative iron transport system according to KEGG mapper analysis (K02013, K02015, K02016). Similarly, KEGG mapper classified the proteins encoded by the gene cluster IGTS8_peg371-374 as iron-siderophore transport proteins (map02010). Another gene cluster (IGTS8_peg2361-2364) encodes homologs of the subunits of the potassium transport Kdp system (KEGG map02020) (61). The expression of these genes was downregulated in the DBT culture during the early-log and stationary phases. However, the relative abundance of the transcripts of the genes encoding the related two-component regulatory system (KdpED) was not significantly different between the DBT and MgSO_4_ cultures (Tables S2 and S6).

The gene cluster IGTS8_peg3566-3570 encodes proteins predicted to constitute a phosphate transport system (Pst) based on KEGG mapper analysis (KEGG map02010). The gene products exhibited high identity to Pst proteins from *Rhodococcus* spp. (Tables S2 and S6). The relative transcript abundance of these genes increased with time and was significantly higher in the DBT culture during the stationary phase (log_2_ fold change = 2.2–4.04). Another predicted phosphate transport system protein is encoded by the IGTS8_peg3558 gene, which was significantly upregulated under biodesulfurization conditions during the late-log and stationary phases (Tables S2 and S6). The predicted function of the IGTS8_peg3558 gene product was confirmed by KEGG mapper analysis (K02029), showing its homology to a phosphate uptake regulator. The gene cluster IGTS8_peg4893-4895 encodes an ABC-type metal transporter homologous to the manganese transport system MntABC, as shown by KEGG mapper analysis (KEGG map02010). UniProt search also showed high identity (76%–80%) of the IGTS8_peg4893-4895 gene products to ABC-type manganese transport proteins from different bacteria (A0ABV2P6Q6, A0ABV2P6Q7, A0A4R6RUY4). These genes were significantly upregulated in the DBT culture during the stationary phase (Tables S2 and S6).

Interestingly, the genome of the IGTS8 strain harbors a gene cluster for [NiFe] hydrogenase maturation and metallocenter assembly proteins (IGTS8_peg1292-1297), which were significantly downregulated in the DBT culture during the early-log phase. Exceptionally, the hydrogenase assembly chaperone HypC/HupF (IGTS8_peg1293) gene was upregulated in the DBT culture during the late-log phase before its expression downshifted in the stationary phase. The genes encoding the hydrogenase subunits (IGTS8_peg1284-1286), which are located upstream of the maturation and assembly genes, exhibited mostly no significant difference in expression levels (Tables S2 and S6). A gene cluster (IGTS8_peg3158-3164) encodes large ribosomal proteins and ribosome hibernation factors. The transcripts of these genes were significantly less abundant under biodesulfurization conditions during the late-log phase. KEGG pathway analysis confirmed the role of the IGTS8_peg3158-3164 gene products as ribosome components (KEGG pathway map number: map03010). Several genes encoding mobile elements (transposons) were also differentially expressed in the biodesulfurizing culture and were mostly upregulated (Table S2).

Biodesulfurization stimulated the differential expression of several genes predicted to encode WhiB family transcriptional regulators. Some of these were upregulated (IGTS8_peg3234), and others were downregulated (IGTS8_peg902, 3276) in the DBT culture depending on the growth phase. By searching the genome of the IGTS8 strain, we found five proteins annotated as WhiB family transcriptional regulators, and all of them are encoded on the chromosome.

### Biodesulfurization is associated with growth rate reduction and morphological changes

To better understand the growth phenotype of the IGTS8 strain under biodesulfurization conditions, it was cultured on either DBT, MgSO_4_, or a mixture of both. As shown in Fig. S8, the three cultures exhibited differences in the growth profile and maximum specific growth rates. The IGTS8 culture grew with approximately a 50% reduction in the specific growth rate when MgSO_4_ was replaced with DBT as the sulfur source. However, a mixture of DBT and MgSO_4_ allowed the cultures to grow slightly slower compared to the MgSO_4_ culture. Thus, indicating that the growth deceleration under biodesulfurization conditions is due to a lack of the preferred sulfur source (inorganic sulfate) and the supply of an organic sulfur compound instead, namely sulfate limitation. Scanning electron microscopy revealed that the DBT culture contained a heterogeneous mixture of cell shapes and sizes, including long rods, short rods, curved cells, irregularly shaped cells, club-shaped cells, as well as cells with multiple divisions. In contrast, the MgSO_4_ culture consisted mainly of short rods and was more homogeneous in terms of cell shapes and sizes (Fig. S9). The average cell size in the DBT culture was 1.17 × 0.66 µm, whereas the MgSO_4_ culture had a relatively smaller average cell size of 0.94 × 0.48 µm.

## DISCUSSION

We recently showed how growth under biodesulfurization conditions can have a profound impact on the proteome and metabolome of the model biodesulfurization strain *R. qingshengii* IGTS8 (31–33). Gene expression profiles are largely consistent with the proteome profile, with some exceptions, indicating that biodesulfurization provokes different responses at the transcriptional and translational levels (30, 41, 62). These deviations are manifested in two aspects, namely, fold change in gene/protein expression and the growth phase effect. The latter was more prominent in the transcriptomic response, particularly during the late-log phase.

It potentially suggests that biodesulfurizing cells maintain homeostasis by exerting a tight posttranscriptional control to ensure fine-tuned production of strictly necessary proteins under the sulfate-limited conditions. This postulation is backed by the observed differential expression of many transcriptional regulators belonging to different protein families (48). Among them, the WhiB family proteins include redox-sensing transcription factors that can be induced by various stimuli or stressors (63–65). A WhiB family protein from *Gordonia* sp. ITTR100 is involved in the repression of the biodesulfurization operon by directly binding to the *dsz* promoter (66). Whether one of those putative WhiB homologs could play a role in the regulation of the *dsz* operon in the strain IGTS8 awaits further studies.

It appears that the transcriptional response of the strain IGTS8 under biodesulfurization conditions prioritized cellular homeostasis and survival rather than growth. This is a common bacterial adaptation strategy under various environmental stressors, as shown for arsenite-treated *Rhodococcus aetherivorans* BCP1 (67) and cold-stressed *Rhodococcus* sp. RCBS9 (48). Under hostile conditions, survival requires energy conservation and deceleration of replication and cell proliferation (48, 68). Therefore, a substantial fraction of the biodesulfurization-associated transcriptome was directed toward energy and resource conservation, cell envelope modification, cellular protection, and growth rate reduction.

Lower growth rate of *R. qingshengii* IGTS8 in the DBT culture, compared to the MgSO_4_ culture, is consistent with the downregulation of glycolytic and TCA cycle enzymes and genes (32, 33, 68), as also reported in *Rhodococcus equi* grown on bisphenol A (69). The strong downshift of the expression of inositol phosphate metabolism genes in the biodesulfurizing culture could be related to metabolic economy, resource availability, and growth phase adaptation. It is also plausible to propose that reducing inositol catabolism could be important to redirect the metabolic flux toward the synthesis of the redox buffer mycothiol or phospholipids (32, 33, 70). This postulation aligns with the reported lower abundance of *myo*-inositol-2-phosphate under biodesulfurization conditions (33). On the contrary to our findings, inositol catabolism genes were upregulated in xylene-grown *Rhodococcus opacus* R7 compared to malate-grown cultures (71).

Downregulation of genes encoding putative chromosome partitioning, septum formation proteins, and large ribosomal proteins agrees with the slower growth rate and proposed downshift in anabolic activities under biodesulfurization conditions (32, 33). In the same context, the much lower abundance of the dihydrofolate reductase gene transcripts in the DBT culture is in alignment with the slower growth rate, thus representing an adaptive response to sulfate limitation stress (72, 73).

The observed morphological changes in the DBT culture suggest cell cycle and division perturbations under biodesulfurization conditions and are consistent with the differential expression of the cell division and cell envelope-related genes (33). Exposure of *Rhodococcus aetherivorans* BCP1 to arsenic oxyanions resulted in differential expression of cell division and cell wall synthesis genes (67). Cell surface modifications represent a key stress response to the hydrophobicity and toxicity of DBT (33, 74, 75) and a protective barrier against several environmental stresses (76). Colanic acid is an anionic heteropolysaccharide produced and secreted by members of *Enterobacteriaceae* and provides protection in hostile environments against several stressors, such as desiccation, oxidative stress, and low pH (76). The colanic acid operon is induced under environmental or cell envelope stress (77). However, colanic acid is unique to Gram-negative bacteria of the *Enterobacteriaceae*, while *Rhodococcus* spp. don’t typically produce it.

Considering sulfate limitation and a higher energy demand in the DBT culture compared to the MgSO_4_ culture (15, 16, 33), upregulation of genes encoding predicted fatty acids, urea, and amino acid degradation enzymes could be reconciled as an adaptation to scavenge energy, reducing equivalents, and anabolic precursors (33, 78). Amino acid metabolism is crucial for pH homeostasis, generation of reducing power, and enhancing resistance to environmental stress (79). Perhaps the upregulation of genes encoding urease and its accessory proteins in the DBT culture during the late-log phase reflects a higher need to detoxify urea and benefit from the degradation products (ammonia) for pH maintenance (80). The importance of pH and ion homeostasis in the DBT culture can also be inferred from the significant overexpression of a putative cation:proton antiporter (81).

A higher abundance of glycine cleavage-related genes in the DBT culture is consistent with the proteomics profiles (33) and suggests a need to avoid glycine inhibition of growth and cell wall biosynthesis under biodesulfurization conditions (33, 82). Moreover, glycine cleavage produces an activated methylene group in the form of 5,10-methylenetetrahydrofolate, which can be utilized in the biosynthesis of purines, pyrimidines, methionine, and S-adenosylmethionine, in addition to NADH, which could be channeled for energy conservation (33, 83). Fatty acid degradation can contribute to acetyl-CoA production (67). In addition, upregulation of fatty acid degradation genes under stress could be a crucial acclimatization strategy for energy conservation and recycling of fatty acids (48, 69). Furthermore, reducing biosynthesis of energetically costly compounds like spermidine conserves energy and metabolic precursors that can be diverted to other critical cellular needs (84).

A general stress response was evident in the DBT culture from the differential expression of related genes (33). For instance, universal stress proteins play a role in sensing environmental stimuli, maintaining enzyme activities, and enhancing cell membrane stability (48, 85). Upregulation of the dipeptide/oligopeptide transport genes in the DBT culture could be an attempt to scavenge sulfur from sulfur-containing peptides as a sulfate limitation response or a general response to nutrient limitation (33, 86, 87). In the same context, increasing the expression of NTA monooxygenases could be an adaptive response to scavenge sulfur from organosulfur compounds (88, 89). A similar finding was reported for *Gordonia* sp. strain NB4-1Y grown on organosulfur compounds versus MgSO_4_ as the sole sulfur source (88).

Furthermore, transcriptional responses in the biodesulfurizing culture suggest the importance of phosphate and metal homeostasis. This could be inferred from the downregulation of genes encoding iron, potassium, and zinc transport systems and the upregulation of genes encoding putative phosphate and manganese uptake proteins. Manganese is known to be crucial for bacterial survival under stress (90). Although the expression level of the genes of the K-sensing signal transduction system (KdpDE) was not significantly different, the genes of the high-affinity ABC-type K transporters were significantly downregulated in the DBT culture (KdpABC). While bacteria typically maintain a very high intracellular concentration of K for osmoregulation, pH homeostasis, and enzyme activity, K uptake is tightly controlled to avoid toxicity when over-accumulated (91). Hence, reducing K uptake under biodesulfurization conditions could protect the cell from additional stresses and/or enable conserving the needed energy for other more important life-supporting processes.

The strong upregulation of the cold shock protein CspA in the DBT culture seems intriguing, as no temperature downshift or cold shock was present in our experiments. However, cold shock proteins are induced not only for cold adaptation but also under other perturbations, including nutrient stress, where they play a role in gene expression. Specifically, cold shock proteins function mainly as RNA chaperones, which bind single-stranded RNA and destabilize secondary structures in mRNA. They also act as transcription antiterminators by preventing hairpin formation (92). Cold shock proteins could be crucial as the culture got older, which explains why the upshift in their expression occurred during the late-log and stationary phases (93).

A higher mutation frequency could enable bacteria to better resist toxic damage by xenobiotics (69, 94). The general stress response favors survival over rapid growth and can regulate the expression of transposases. Therefore, upregulation of genes encoding transposases often occurs as a stress response and to enable genetic adaptation and genome plasticity. This may explain why the expression of several genes encoding mobile genetic elements was higher under biodesulfurization conditions. A higher level of transposases can increase transposition events, leading to higher mutation rates and genome rearrangements, resulting in rapid adaptation. Through these mutations, the stressed cells may acquire some beneficial traits that can relieve the stress (95–97).

Upregulation of a carbonic anhydrase-encoding gene in the DBT culture suggests a role under biodesulfurization conditions. Carbonic anhydrases are common to all domains of life, and they are involved in various essential physiological processes. The latter include maintaining acid-base homeostasis, regulation of pH and fluid balance, CO_2_ transport and fixation, respiration, photosynthesis, and metabolism of xenobiotics (98, 99). Recently, proteomics and transcriptomics studies on *Gordonia alkanivorans* 135 revealed upregulation of a carbonic anhydrase in a culture grown on DBT as the sulfur source compared to a sulfate-grown culture (30). The authors proposed that carbonic anhydrase could be important for pH homeostasis in the desulfurizing culture. A similar role could be reconciled for the biodesulfurizing strain IGTS8. Alternatively, we propose a role in CO_2_ sensing and carbon fixation, aligning with the upregulation of putative CO dehydrogenase and C1 metabolism-related genes and enzymes (33).

One of the intriguing findings of the transcriptomic profile is the upregulation of nitrile hydratase genes during sulfur acquisition from DBT. Nitrile hydratases can have key functions in detoxification and stress adaptation, which could be beneficial in the biodesulfurizing cells (46–48). Nonetheless, we could not detect any nitrile hydratases in the proteome of the strain IGTS8 (35), which raises the question of why the nitrile hydratase genes were upregulated in the DBT culture. The ability of the strain IGTS8 to grow on acetonitrile as the sole carbon source indicates the presence and expression of nitrile hydratase genes. However, their role under biodesulfurization conditions remains to be elucidated.

While the transcriptomic profile suggests a robust global response to DBT, we acknowledge that the functional prediction for the gene products relies largely on homology, a common limitation of systems biology studies. Hence, gene knockout, complementation, and phenotypic characterization studies are still needed to conclusively reveal the roles of stress response proteins identified here under biodesulfurization conditions. Although the transcriptional responses suggest main directions of the physiological and metabolic adaptations of the biodesulfurizing strain IGTS8, the presence of a large number of genes encoding hypothetical proteins among the highly differentially expressed genes represents a significant limitation of the RNA-seq analysis. Previous transcriptomic profiling studies on several *Rhodococcus* strains reported similar findings under different stress conditions (65, 71, 88, 100, 101). Looking at the upside of this limitation, it appears that biodesulfurization-associated physiological and metabolic adaptations of the strain IGTS8 go far beyond the transcriptional responses reported here. Therefore, identification and characterization of these hypothetical proteins will reveal novel aspects not only of the biodesulfurization phenotype but also of physiology, metabolism, and stress response of *Rhodococcus* spp. in general. Nonetheless, our findings highlight some gene targets for metabolic engineering and synthetic biology studies that could be designed to develop more efficient biodesulfurizing strains. These future metabolic engineering endeavors should overcome growth and cell division restrictions, enable better energy supply, and support intracellular homeostasis under biodesulfurization conditions.

### Conclusions

Biodesulfurization is perceived as a stress signal that provokes a global transcriptomic response, generally aligning with the recently reported proteomic rearrangements. The stress response encompasses both sulfate-limitation specific, as well as general adaptations, and reaches far beyond the common sulfate starvation-induced response. Although the transcriptomic rearrangements were largely growth phase-dependent, they were mainly orchestrated to optimize cell protection, homeostasis, and resource management, while reducing growth and anabolic activities. Further adaptive responses under biodesulfurization conditions will be revealed as the identity and function of the detected hypothetical proteins encoded in the IGTS8 genome continue to be revealed in the future.

## Data Availability

Data that support the findings of this study are openly available in the SRA under BioProject accession number PRJNA1311948.

## SUPPLEMENTAL MATERIAL

Supplemental material aem.01549-26-s0001.pdf Supplemental methods; Tables S1, S3, S4, and S6; Fig. S1 to S9.

Table S2 aem.01549-26-s0002.xlsx Differential analysis of the RNA-seq data.

Table S5 aem.01549-26-s0003.xlsx qPCR results and differential analysis.

ASM does not own the copyrights to Supplemental Material that may be linked to, or accessed through, an article. The authors have granted ASM a non-exclusive, world-wide license to publish the Supplemental Material files. Please contact the corresponding author directly for reuse.
