# Supplementary material for: Sulfur source-driven global remodeling of gene expression in the fuel-biodesulfurizing Rhodococcus qingshengii IGTS8
Source: Appl Environ Microbiol. 2026 Sep 23;92(10):e01549-26. doi: 10.1128/aem.01549-26 (PMC13689224; doi:10.1128/aem.01549-26)
Supplement: Supplementary file 1 [file aem.01549-26-s0001.pdf]

# Supplementary Material

## Applied and Environmental Microbiology

# Sulfur Source-Driven Global Remodeling of Gene Expression in the Fuel-Biodesulfurizing *Rhodococcus qingshengii* IGTS8

Ashraf El Nayal[a], Vartul Sangal[b], Abdelmalek Alioua[c], Aurélie Hirschler[d], Sandrine Koechler[c], Dimitri Heintz[e], Christine Carapito[d], Alain Van Dorsselaer[d] ,Wael Ismail[a]*

[a]Center of Environmental and Biological Studies, Arabian Gulf University, Bahrain

[b]School of Geography and Natural Sciences, Northumbria University, Newcastle upon Tyne, United Kingdom

[c]Institut de biologie de moléculaire des plantes CNRS, 12 rue du général Zimmer, Strasbourg, France

[d]Laboratoire de Spectrométrie de Masse BioOrganique, CNRS, Université de Strasbourg, IPHC UMR 7178, Infrastructure Nationale de Protéomique ProFI - UAR2048, Strasbourg, France.

[e]Université de Strasbourg, CNRS, IPHC UMR 7178, 23 rue du Loess, F-67000 Strasbourg, France

*Corresponding author and lead contact: Center of Environmental and Biological Studies, Arabian Gulf University, Bahrain, Tel: +97336146948, e-mail: waelame@agu.edu.bh

## Supplementary methods

### Scanning electron microscopy

The electron microscopy work was conducted at ExpressMed Diagnostics and Research (Bahrain). Culture samples were treated with 3% glutaraldehyde in phosphate buffer for 2 hours at 4°C, followed by rinsing 10 times with phosphate buffer to remove residual fixative. Gradual dehydration was performed using an ethanol series (1× each-10 min: 30%, 50%, 70%, 90%., 3× at 100%-10 min, with 5-10 min per step). The samples were manually treated with hexamethyldisilazane (HMDS) for 10 min for drying and then were placed on a carbon adhesive tape attached to aluminum stubs. The bacterial surface was coated with ~8 nm gold layer using a Leica EM ACE600 sputter coater (Leica-Microsystems, Germany). Bacterial images were captured using Thermofisher Scientific-USA, Quattro S FESEM (Field Emission Scanning Electron Microscopy) at 10KV and various magnifications.

Supplementary Table S1: A list of genes included in the qPCR validation.

| Gene ID | Annotation | Expression Profile in the DBT culture |
|---|---|---|
| IGTS8_peg2666 | Succinate dehydrogenase flavoprotein subunit (EC 1.3.99.1) | Upregulated |
| IGTS8_peg3410 | putative monooxygenase | Upregulated |
| pIGTS8_peg6353 | AcylCoAdehydrogenase; probable dibenzothiophene desulfurization enzyme | Upregulated |
| IGTS8_peg5782 | Amino acid ABC transporter permease protein | Upregulated |
| IGTS8_peg5940 | Possible transcriptional regulator, ROK family | Upregulated |
| IGTS8_peg3803 | Possible Linoleoyl-CoA desaturaee (Delta(6)-desaturase) | Upregulated |
| IGTS8_peg4198 | Transcriptional regulator, LuxR family | Upregulated |
| IGTS8_peg5812 | Thiazole biosynthesis protein ThiG | Upregulated |
| IGTS8_peg2085 | Glycogen debranching enzyme (EC 3.2.1.-) | Downregulated |
| IGTS8_peg4040 | Gluconate transporter family protein | Downregulated |
| IGTS8_peg781 | Dihydrofolate reductase (EC 1.5.1.3) | Downregulated |
| IGTS8_peg4192 | Kynurenine formamidase, bacterial (EC 3.5.1.9) | Downregulated |
| pIGTS8_peg6267 | Hypothetical protein | Downregulated |
| pIGTS8_peg6548 | Hypothetical protein | Downregulated |
| pIGTS8_peg6648 | Hypothetical protein | Downregulated |
| IGTS8_peg141 | Hypothetical protein | Downregulated |

Supplementary Table S3: Significantly overexpressed genes in the DBT culture commonly found in cells from all growth phases.

| **Gene ID** | **Annotation** | **Proposed Function/Pathway** |
| --- | --- | --- |
| IGTS8_peg1895 | FIG00997377: Hypothetical protein | |
| IGTS8_peg3750 | Predicted transcriptional regulator | MarR family winged helix-turn-helix transcriptional regulator |
| IGTS8_peg3751 | Hypothetical protein | |
| IGTS8_peg4573 | Transcriptional regulator | Transcriptional regulator |
| IGTS8_peg4574 | Hypothetical protein | |
| IGTS8_peg4575 | $Na^+/H^+$ Antiporter | Cation:proton antiporter |

Supplementary Table S4: Significantly downregulated genes in the DBT culture commonly found in cells from all growth phases.

| Gene ID | Annotation | Proposed Function/Pathway |
|---|---|---|
| IGTS8_peg781 | Dihydrofolate reductase (EC 1.5.1.3) | Folate biosynthesis |
| IGTS8_peg782 | Methylmalonate-semialdehyde dehydrogenase [inositol] (EC 1.2.1.27) | Carbon and amino acid metabolism |
| IGTS8_peg783 | Glyceraldehyde-3-phosphate ketol-isomerase (EC 5.3.1.1) | myo-Inositol catabolism protein IolH |
| IGTS8_peg784 | Myo-inositol 2-dehydrogenase (EC 1.1.1.18) | myo-Inositol catabolism |
| IGTS8_peg785 | Inosose dehydratase (EC 4.2.1.44) | Inositol phosphate metabolism |
| IGTS8_peg786 | Epi-inositol hydrolase (EC 3.7.1.-) | Inositol phosphate metabolism |
| IGTS8_peg787 | 5-Deoxy-glucuronate isomerase (EC 5.3.1.-) | Inositol phosphate metabolism |
| IGTS8_peg788 | 5-keto-2-deoxy-D-gluconate-6 phosphate aldolase [form 2] (EC 4.1.2.29) | Inositol phosphate metabolism |
| IGTS8_peg789 | 5-keto-2-deoxygluconokinase (EC 2.7.1.92) | Inositol phosphate metabolism |
| IGTS8_peg790 | Transcriptional regulator, GntR family | Transcriptional regulator |
| IGTS8_peg791 | Myo-inositol 2-dehydrogenase (EC 1.1.1.18) | myo-Inositol catabolism |
| IGTS8_peg800 | Inositol transport system sugar-binding protein | Inositol Transport |
| IGTS8_peg801 | Inositol transport system permease protein | Inositol Transport |
| IGTS8_peg802 | Inositol transport system ATP-binding protein | Inositol Transport |
| IGTS8_peg2230 | Hypothetical protein | Uncharacterized protein |
| IGTS8_peg2231 | Putative cytochrome P450 hydroxylase | Hydroxylase |
| IGTS8_peg2232 | Hypothetical protein | UbiA family prenyltransferase |
| IGTS8_peg2233 | Hypothetical protein | Acyl carrier protein |
| IGTS8_peg2234 | 3-oxoacyl-[acyl-carrier-protein] synthase, KASII (EC 2.3.1.41) | Ketosynthase family 3 (KS3) domain-containing protein |

| | | |
|---|---|---|
| IGTS8_peg2235 | Hypothetical protein | Extracellular membrane protein CFEM domain-containing protein |
| IGTS8_peg2236 | Benzoate-CoA ligase (EC 6.2.1.25) | AB hydrolase-1 domain-containing protein |
| IGTS8_peg2237 | Putative transmembrane efflux protein | Major facilitator superfamily (MFS) transporter facilitates the transport across cytoplasmic or internal membranes of one or more from a variety of substrates including ions, sugar phosphates, drugs, neurotransmitters, nucleosides, amino acids, and peptides |
| IGTS8_peg2238 | Hypothetical protein | major facilitator superfamily (MFS) transporter |
| IGTS8_peg2239 | Putative geranylgeranyl pyrophosphate synthase | Polyprenyl synthetase family protein |
| IGTS8_peg2240 | Hypothetical protein | major facilitator superfamily (MFS) transporter |
| IGTS8_peg4004 | Hypothetical protein | Sortase |
| IGTS8_peg4005 | TolA protein | Cell surface protein |

Supplementary Table S6: Functional prediction for differentially expressed genes

| Gene ID | Annotation | Close matches (accession no.) | % Identity | E-value | Pathway/Proposed Function or Functional Category |
|---|---|---|---|---|---|
| IGTS8_peg151 | Major facilitator superfamily MFS_1 | MFS transporter [Rhodococcus qingshengii] (WP_083058043.1) | 98 | 0.0 | Iron transport |
| IGTS8_peg152 | ABC transporter (iron.B12.siderophore.hemin) permease component | iron ABC transporter permease [Rhodococcus sp. (in: high G+C Gram-positive bacteria)] (MDN5545993.1) | 96 | 0.0 | Iron transport |
| IGTS8_peg153 | Iron compound ABC transporter, ATP-binding protein | ABC transporter ATP-binding protein [Rhodococcus] (WP_020968054.1) | 99 | 0.0 | Iron transport |
| IGTS8_peg154 | Vitamin B12 ABC transporter, B12-binding component BtuF | ABC transporter substrate-binding protein [Rhodococcus sp. 2.95] (MGE2847285.1) | 99 | 0.0 | Iron transport |
| IGTS8_peg170 | Sulfotransferase | trehalose 2-sulfotransferase [Rhodococcus erythropolis] (WP_095969418.1) | 99 | 6e-179 | Biosynthesis of sulfated metabolites |
| IGTS8_peg800 | Inositol transport system sugar-binding protein | substrate-binding domain-containing protein [Rhodococcus sp. NPDC056516] (WP_378636315.1) | 99 | 0.0 | Putative sugar transporter |
| IGTS8_peg801 | Inositol transport system permease protein | ABC transporter permease [Rhodococcus] (WP_069145865.1) | 99 | 0.0 | Putative sugar transporter |
| IGTS8_peg802 | Inositol transport system ATP-binding protein | ATP-binding cassette domain-containing protein [Actinomycetota bacterium] (MHK7370651.1) | 99 | 0.0 | Putative sugar transporter |
| IGTS8_peg1284 | [NiFe] hydrogenase nickel incorporation-associated protein HypB | hydrogenase nickel incorporation protein HypB, partial [Rhodococcus erythropolis] (WP_225989563.1) | 96 | 2e-149 | Hydrogenase |
| IGTS8_peg1285 | Uptake hydrogenase small subunit precursor | hydrogenase accessory protein HypB [Rhodococcus sp. (in: high G+C Gram-positive bacteria)] (RZL75798.1) | 90 | 0.0 | Hydrogenase |
| IGTS8_peg1286 | Uptake hydrogenase large subunit | nickel-dependent hydrogenase large subunit [unclassified Rhodococcus (in: high G+C Gram-positive bacteria)] (WP_378425996.1) | 92 | 0.0 | Hydrogenase |
| IGTS8_peg1292 | Hydrogenase maturation protease | hydrogenase maturation protease [Rhodococcus] (WP_166809607.1) | 93 | 1e-102 | Hydrogenase formation and assembly |
| IGTS8_peg1293 | Hydrogenase assembly chaperone HypC/HupF | HypC/HybG/HupF family hydrogenase formation chaperone [Rhodococcus qingshengii] (WP_373938382.1) | 97 | 3e-57 | Hydrogenase formation and assembly |
| IGTS8_peg1294 | [NiFe] hydrogenase metallocenter assembly protein HypF | carbamoyltransferase HypF [Rhodococcus] (WP_070161853.1) | 99 | 0.0 | Hydrogenase formation and assembly |

| | | | | | |
|---|---|---|---|---|---|
| IGTS8_peg1295 | [NiFe] hydrogenase metallocenter assembly protein HypC | HypC/HybG/HupF family hydrogenase formation chaperone [Rhodococcus sp. IEGM 1409] (WP_283303864.1) | 99 | 4e-63 | Hydrogenase formation and assembly |
| IGTS8_peg1296 | [NiFe] hydrogenase metallocenter assembly protein HypD | hydrogenase formation protein HypD [Rhodococcus sp. ARC_M6] (WP_243621485.1) | 96 | 0.0 | Hydrogenase formation and assembly |
| IGTS8_peg1297 | [NiFe] hydrogenase metallocenter assembly protein HypE | hydrogenase expression/formation protein HypE [Rhodococcus sp. NPDC056506] (WP_378649472.1) | 96 | 0.0 | Hydrogenase formation and assembly |
| IGTS8_peg1817 | Glycine dehydrogenase [decarboxylating] (glycine cleavage system P protein) (EC 1.4.4.2) | aminomethyl-transferring glycine dehydrogenase [Rhodococcus sp.] | 99 | 0.0 | Glycine degradation |
| IGTS8_peg2140 | rRNA small subunit methyltransferase H | 16S rRNA (cytosine(1402)-N(4))-methyltransferase RsmH [Rhodococcus sp. NPDC056506] (WP_378647155.1) | 99 | 0.0 | rRNA methylation/ribosome biogenesis |
| IGTS8_peg2141 | Cell division protein MraZ | division/cell wall cluster transcriptional repressor MraZ [Rhodococcus sp. 5A-K4] (WP_408989271.1) | 99 | 2e-99 | Cell division/cell wall cluster transcription factor |
| IGTS8_peg2303 | Regulator of polyketide synthase expression | PucR family transcriptional regulator [Rhodococcus sp. NPDC059969] (WP_378546931.1) | 99 | 0.0 | Lipid metabolism |
| IGTS8_peg2304 | Malonyl CoA-acyl carrier protein transacylase | ACP S-malonyltransferase [Rhodococcus sp. IEGM 1241] (WP_317830781.1) | 99 | 0.0 | Lipid metabolism |
| IGTS8_peg2305 | Acyl carrier protein | meromycolate extension acyl carrier protein AcpM [Rhodococcus] (WP_033233096.1) | 98 | 2e-54 | Lipid metabolism |
| IGTS8_peg2306 | 3-oxoacyl-[acyl-carrier-protein] synthase, KASII | KasA/KasB family beta-ketoacyl-ACP synthase [Rhodococcus] (WP_020969930.1) | 99 | 0.0 | Lipid metabolism/mycolic acid biosynthesis |
| IGTS8_peg2307 | Propionyl-CoA carboxylase beta chain | acyl-CoA carboxylase subunit beta [Rhodococcus erythropolis] (WP_020908155.1) | 99 | 0.0 | Lipid and carbon metabolism |
| IGTS8_peg2359 | DNA-binding response regulator KdpE | Transcriptional regulatory protein KdpE (Rhodococcus qingshengii JCM 15477) (A0AB38R7N2) | 100 | 4.9e-158 | Potassium transport |
| IGTS8_peg2360 | Osmosensitive K+ channel histidine kinase KdpD | histidine kinase (Rhodococcus qingshengii JCM 15477) (A0AB38R6N1) | 99.9 | 0.0 | Potassium transport |
| IGTS8_peg2361 | Potassium-transporting ATPase C chain (EC 3.6.3.12) | Potassium-transporting ATPase KdpC subunit (Rhodococcus qingshengii JCM 15477) (A0AB38R6N0) | 100 | 6.8e-151 | Potassium transport |
| IGTS8_peg2362 | Potassium-transporting ATPase B chain (EC 3.6.3.12) | Potassium-transporting ATPase ATP-binding subunit kdpB (Rhodococcus baikonurensis) (A0ABV5X9G8) | 100 | 5e-67 | Potassium transport |
| IGTS8_peg2363 | Potassium-transporting ATPase B chain (EC 3.6.3.12) | Potassium-transporting ATPase ATP-binding subunit kdpB | 100 | 0.0 | Potassium transport |

| | | | | | |
|---|---|---|---|---|---|
| | | (Rhodococcus qingshengii JCM 15477) (A0AB38R711) | | | |
| IGTS8_peg2364 | Potassium-transporting ATPase A chain (EC 3.6.3.12) | Potassium-transporting ATPase potassium-binding subunit kdpA (Rhodococcus erythropolis (strain PR4 / NBRC 100887) (C1A1E9) | 99 | 0.0 | Potassium transport |
| IGTS8_peg3307 | Organic hydroperoxide resistance transcriptional regulator | MarR family winged helix-turn-helix transcriptional regulator [Rhodococcus sp. OK302] (WP_094271475.1) | 91 | 5e-94 | Transcription factor/organic hydroperoxide resistance regulator |
| IGTS8_peg3558 | Phosphate transport system regulatory protein PhoU | phosphate signaling complex protein PhoU [Rhodococcus] Sequence ID: WP_037230533.1 | 94 | 7e-153 | Phosphate uptake regulator PhoU [Inorganic ion transport and metabolism] |
| IGTS8_peg3566 | Phosphate transport ATP-binding protein PstB (TC 3.A.1.7.1) | Phosphate ABC transporter ATP-binding protein PstB (Rhodococcus baikonurensis) (A0ABV5XHW7) | 100 | 0.0 | Phosphate transport |
| IGTS8_peg3567 | Phosphate transport system permease protein PstA (TC 3.A.1.7.1) | Phosphate transport system permease protein PstA (Rhodococcus baikonurensis) (A0ABV5XHU6) | 100 | 0.0 | Phosphate transport |
| IGTS8_peg3568 | Phosphate transport system permease protein PstC (TC 3.A.1.7.1) | Phosphate transport system permease protein PsTC (Rhodococcus qingshengii JCM 15477) (A0AB38R9G9) | 100 | 0.0 | Phosphate transport |
| IGTS8_peg3570 | Phosphate ABC transporter, periplasmic phosphate-binding protein PstS (TC 3.A.1.7.1) | phosphate ABC transporter substrate-binding protein PstS [Rhodococcus erythropolis] (WP_269577149.1) | 99 | 0.0 | Phosphate transport |
| IGTS8_peg3887 | OsmC family protein | OsmC family protein [Rhodococcus sp. IEGM 1409] (WP_283302906.1) | 97 | 2e-116 | Peroxiredoxin/Oxidative stress response |
| IGTS8_peg3888 | O-acetylhomoserine sulfhydrylase | bifunctional o-acetylhomoserine/o-acetylserine sulfhydrylase [Rhodococcus sp. 27YEA15] (WP_424808574.1) | 93 | 0.0 | Cysteine and methionine biosynthesis |
| IGTS8_peg4215 | COG3173: Predicted aminoglycoside phosphotransferase | phosphotransferase family protein [Rhodococcus sp. AD45-ID] (WP_045065460.1) | 97 | 0.0 | Antibiotic resistance |
| IGTS8_peg4389 | Glycosyltransferase | glycosyltransferase [Rhodococcus qingshengii] (WP_208721950.1) | 100 | 0.0 | synthesize oligosaccharides, polysaccharides, and glycoconjugates |
| IGTS8_peg4431 | Cold shock protein CspA | cold-shock protein [Rhodococcus qingshengii] (WP_221742973.1) | 98 | 1e-59 | Cold stress response |
| IGTS8_peg4444 | Universal stress protein family | universal stress protein [Rhodococcus sp. 1139] (WP_070161685.1) | 89 | 5e-178 | General stress response |
| IGTS8_peg5580 | Alkaline shock protein 23 | Asp23/Gls24 family envelope stress response protein [unclassified Rhodococcus (WP_378535915.1) | 97 | 7e-98 | Cell envelope stress |

| | | | | | |
|---|---|---|---|---|---|
| IGTS8_peg5599 | Undecaprenyl-phosphate galactosephosphotransferase (EC 2.7.8.6) | sugar transferase [Rhodococcus qingshengii] (WP_003943685.1) | 99 | 0.0 | Sugar transferase, exopolysaccharide (lipopolysaccharides and colonic acid) biosynthesis |
| IGTS8_peg5600 | hypothetical protein | glycosyltransferase [Rhodococcus] (WP_065456022.1) | 99 | 0.0 | Glycosyl transferase, cell wall biosynthesis |
| IGTS8_peg5601 | hypothetical protein | polysaccharide pyruvyl transferase family protein [Rhodococcus sp. UFZ-B548] (WP_259349101.1) | 96 | 0.0 | Polysaccharide pyruvyl transferase |
| IGTS8_peg5602 | hypothetical protein | O-antigen ligase family protein [Rhodococcus erythropolis] (WP_317558241.1) | 95 | 0.0 | O-antigen ligase family protein, exopolysaccharide production |
| IGTS8_peg5603 | Capsular polysaccharide synthesis enzyme CpsI, glycosyltransferase | glycosyltransferase [Rhodococcus qingshengii] (WP_042452284.1) | 99 | 0.0 | Glycosyl transferase, Capsular polysaccharide synthesis, involved in cell wall biosynthesis |
| IGTS8_peg5604 | Colanic acid biosynthesis glycosyl transferase WcaE | glycosyltransferase [Rhodococcus sp. IC4_135] (WP_166812207.1) | 95 | 3e-180 | Glycosyl transferase, involved in cell wall biosynthesis |
| IGTS8_peg5606 | hypothetical protein | glycosyltransferase family 2 protein [Rhodococcus qingshengii] (WP_083056152.1) | 100 | 4e-156 | Glycosyl transferase |
| IGTS8_peg5607 | putative integral membrane protein | lipopolysaccharide biosynthesis protein [Rhodococcus erythropolis] (WP_084325750.1) | 96 | 0.0 | Polysaccharide biosynthesis protein, O-antigen/teichoic acid export membrane protein, Oligosaccharide flippase family protein |
| IGTS8_peg5608 | GDP-mannose 4,6-dehydratase (EC 4.2.1.47) | GDP-mannose 4,6-dehydratase [Rhodococcus erythropolis] (WP_317552703.1) | 90 | 0.0 | Fucose biosynthesis |
| IGTS8_peg5609 | GDP-L-fucose synthetase (EC 1.1.1.271) | GDP-L-fucose synthase family protein [unclassified Rhodococcus (in: high G+C Gram-positive bacteria)] (WP_283376861.1) | 98 | 0.0 | Fucose biosynthesis |
| IGTS8_peg5610 | UDP-glucose dehydrogenase (EC 1.1.1.22) | UDP-glucose dehydrogenase family protein [Rhodococcus sp. NPDC056506] (WP_378645703.1) | 92 | 0.0 | Fucose biosynthesis |
| IGTS8_peg5611 | Tyrosine-protein kinase Wzc (EC 2.7.10.2) | tyrosine-protein kinase domain-containing protein [Rhodococcus sp. NPDC057135] (WP_378448597.1) | 95 | 0.0 | Polysaccharide biosynthesis tyrosine autokinase |

| | | | | | |
|---|---|---|---|---|---|
| IGTS8_peg5905 | Isocitrate lyase (EC 4.1.3.1) | isocitrate lyase [Rhodococcus olei] ( WP_345350381.1) | 94 | 0.0 | Isocitrate lyase |
| IGTS8_peg5907 | 5-methyltetrahydropteroyltriglutamate-- homocysteine methyltransferase (EC 2.1.1.14) | 5-methyltetrahydropteroyltriglutamate--homocysteine S-methyltransferase [Rhodococcus] (WP_073512145.1) | 99 | 0.0 | Cysteine and methionine biosynthesis |
| IGTS8_peg5912 | anti-sigma factor antagonist | STAS domain-containing protein [Rhodococcus erythropolis] (WP_307846847.1) | 99 | 1e-66 | STAS (Sulphate Transporter and AntiSigma factor antagonist |
| IGTS8_peg997 | Carbonic anhydrase (EC 4.2.1.1) | beta-class carbonic anhydrase [Rhodococcus sp. 27YEA15] (WP_424807433.1) | 92 | 4e-109 | Carbon utilization/Energy metabolism |
| IGTS8_peg5020 | Carbonic anhydrase (EC 4.2.1.1) | carbonic anhydrase [Rhodococcus sp. IEGM 1379] (WP_283263294.1) | 92 | 6e-137 | Carbon utilization /Energy metabolism |
| IGTS8_peg3341 | Carbonic anhydrase (EC 4.2.1.1) | SulP family inorganic anion transporter [Rhodococcus erythropolis] (WP_092763049.1) | 99 | 0.0 | Carbon utilization /Energy metabolism/Transmembrane transport |
| IGTS8_peg5608 | GDP-mannose 4,6-dehydratase (EC 4.2.1.47) | GDP-mannose 4,6-dehydratase [Rhodococcus erythropolis] (WP_317552703.1) | 90 | 0.0 | Nucleotide sugar biosynthesis |
| IGTS8_peg5609 | GDP-L-fucose synthetase (EC 1.1.1.271) | GDP-L-fucose synthase family protein [unclassified Rhodococcus] (WP_283376861.1) | 98 | 0.0 | Nucleotide sugar biosynthesis |
| IGTS8_peg5610 | UDP-glucose dehydrogenase (EC 1.1.1.22) | UDP-glucose dehydrogenase family protein [Rhodococcus sp. NPDC056506] (WP_378645703.1) | 92 | 0.0 | Nucleotide sugar biosynthesis |
| IGTS8_peg5867 | Polyphosphate kinase 2 (EC 2.7.4.1) | polyphosphate kinase 2 [Rhodococcus erythropolis] (MDF2468726.1) | 100 | 0.0 | Energy metabolism |
| pIGTS8_peg6638 | Prevent host death protein, Phd antitoxin | YlcI/YnfO family protein [Rhodococcus sp. NPDC057135] (WP_378449875.1) | 96 | 2e-44 | Antitoxin component of a type II toxin-antitoxin (TA) system |
| pIGTS8_peg6682 | Programmed cell death toxin YdcE | type II toxin-antitoxin system PemK/MazF family toxin [Rhodococcus erythropolis] (WP_030003057.1) | 94 | 3e-71 | mRNA-degrading endonuclease MazF, toxin component of the MazEF toxin-antitoxin module |

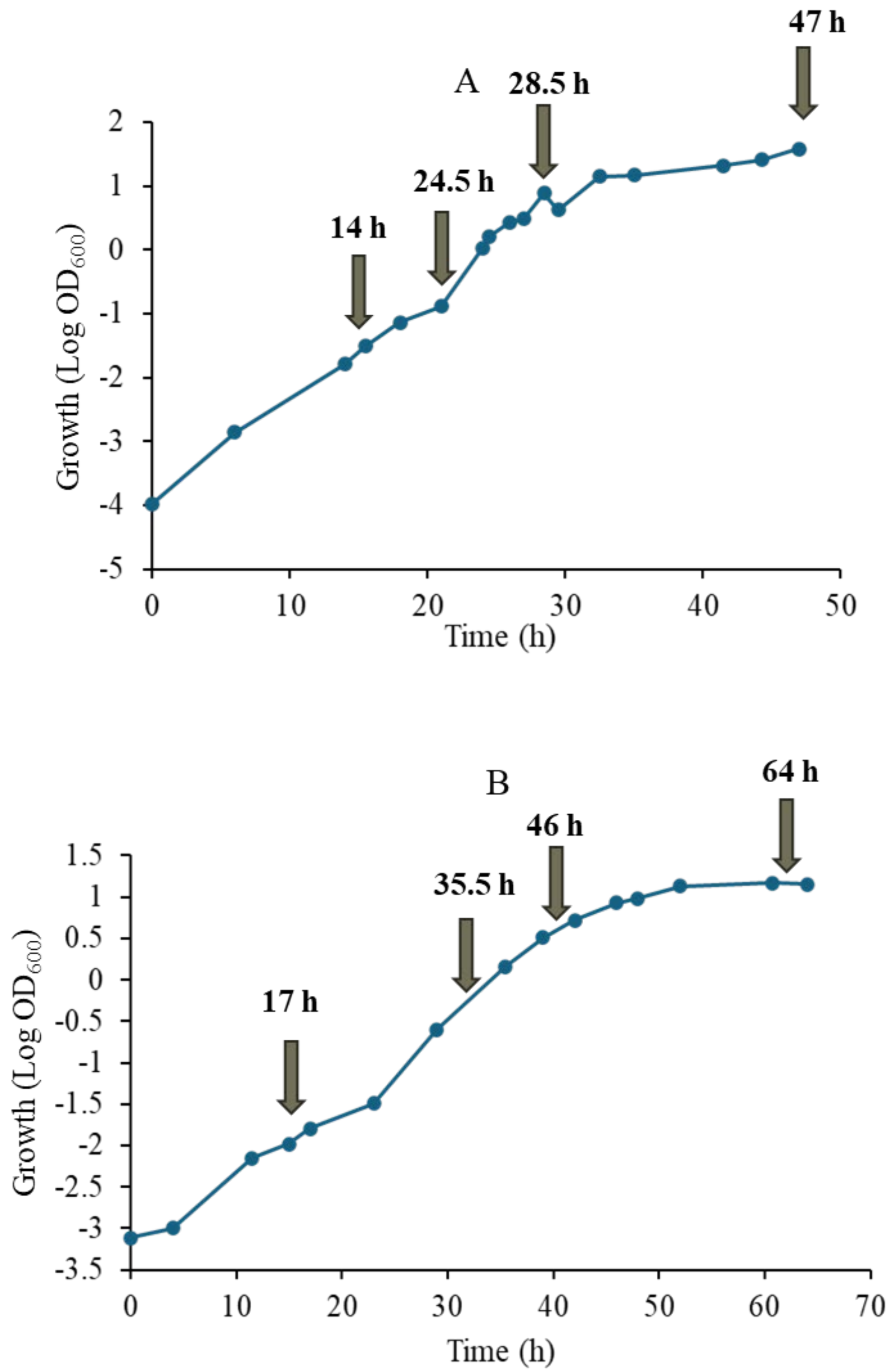


**Figure S1:** Growth profiles of *R. qingshengii* IGTS8 cultures on 0.5 mM of either $MgSO_4$ (A) or DBT (B) as the sulfur source and 20 mM glucose + 85.7 mM ethanol as the carbon source. All cultures were grown in chemically defined medium and cells were harvested at time intervals as shown by the arrows. All values are average of four biological replicates and growth is shown on a logarithmic scale. These cultures were used for the transcriptional profiling.

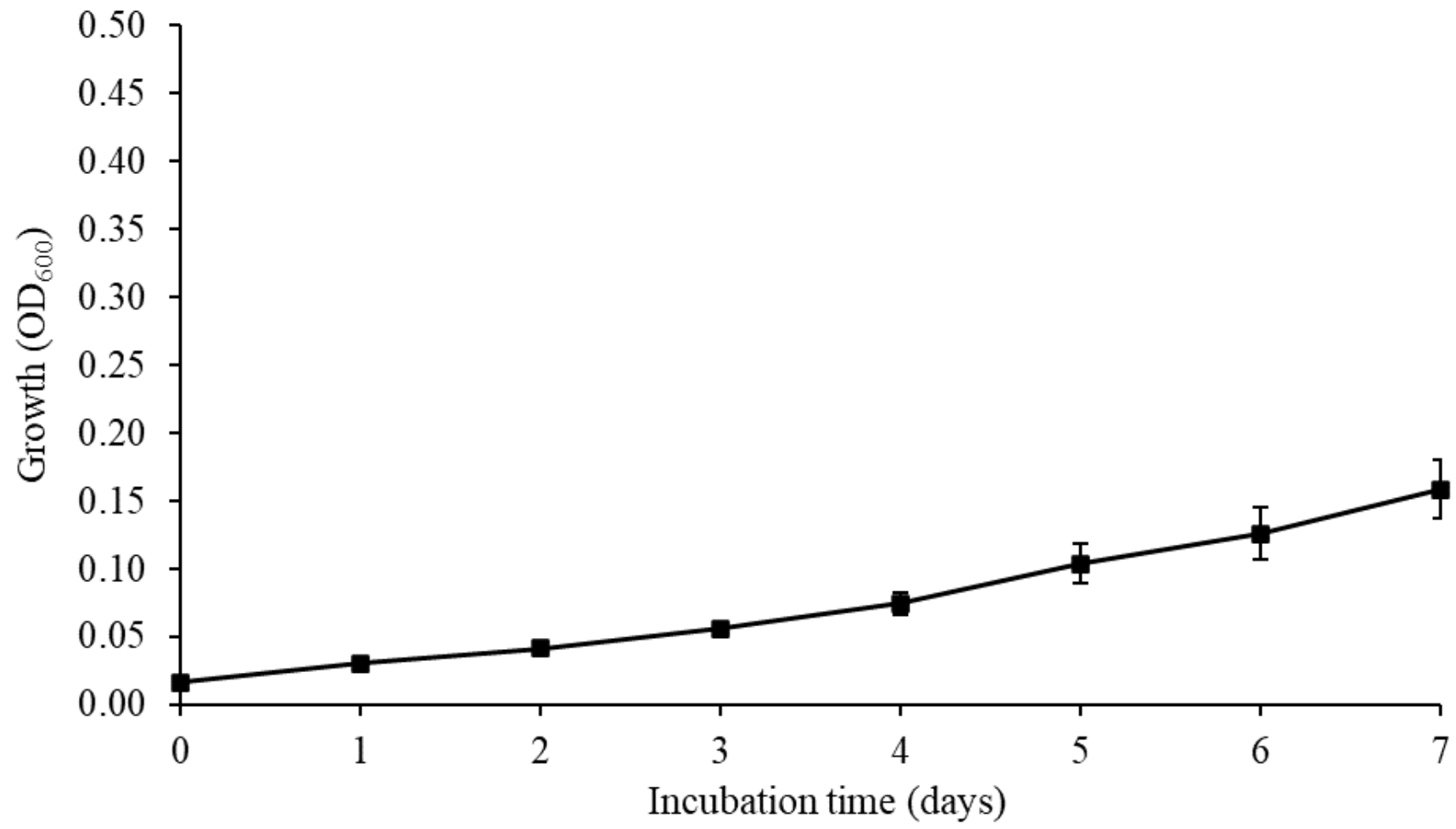


**Figure S2:** Growth of *R. qingshengii* IGTS8 in CDM containing glucose + ethanol as the carbon source without addition of sulfur sources (neither DBT nor $MgSO_4$). All values are average of three biological replicates.

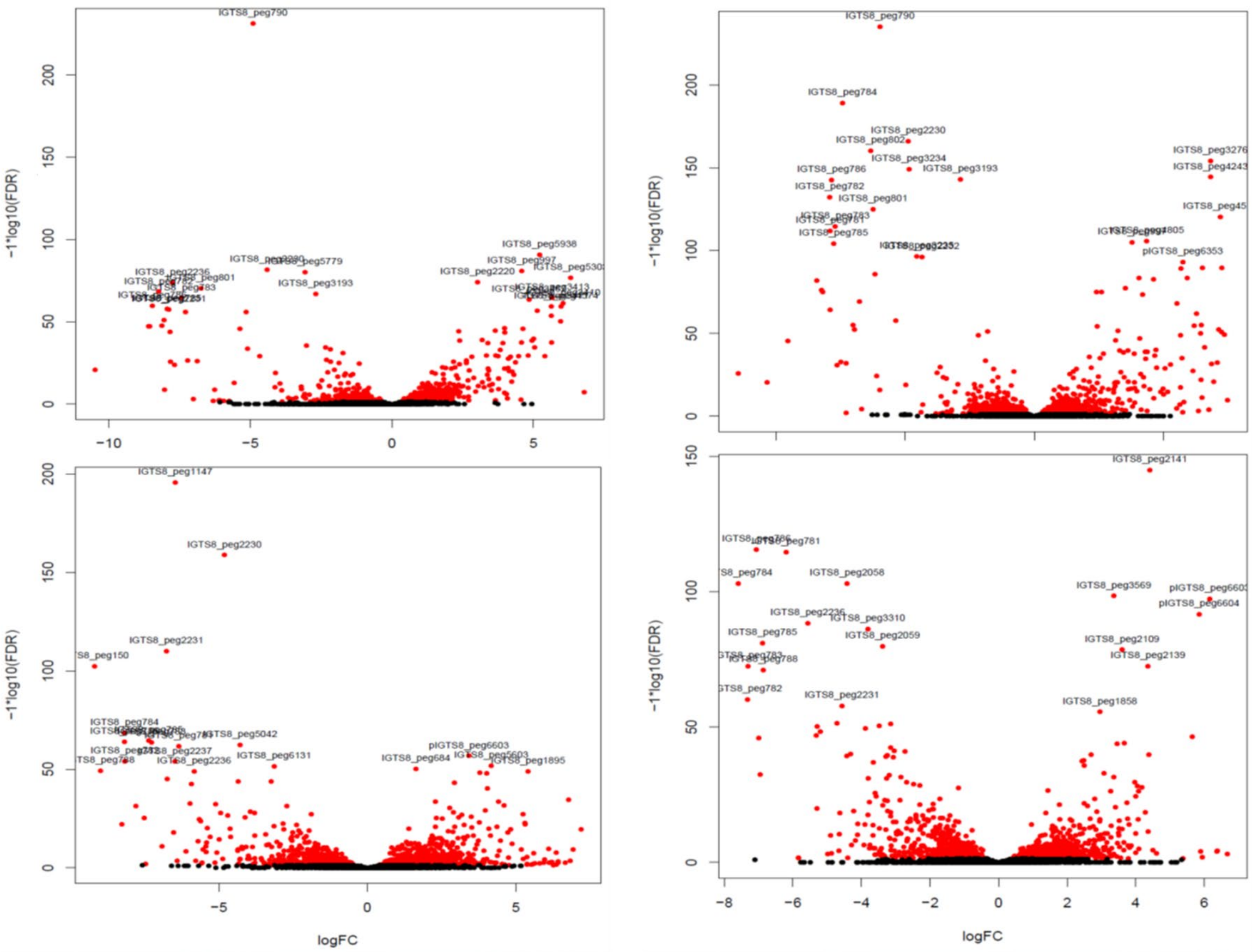


**Figure S3:** Volcano plots showing differential gene expression under biodesulfurization versus inorganic sulfur conditions in (i) early-log, (ii) mid-log, (iii) late-log and (iv) stationary phases. The x-axis represents $\log_2$-fold change (logFC), and the y-axis represents $-\log_{10}$(FDR-adjusted *p*-value). Each point represents an individual gene. Red points indicate genes with significant differential expression (FDR < 0.05), while black points represent genes with no significant change. Selected genes with the highest statistical significance are labeled. Negative logFC values indicate downregulation under biodesulfurization conditions and positive values indicate upregulation.

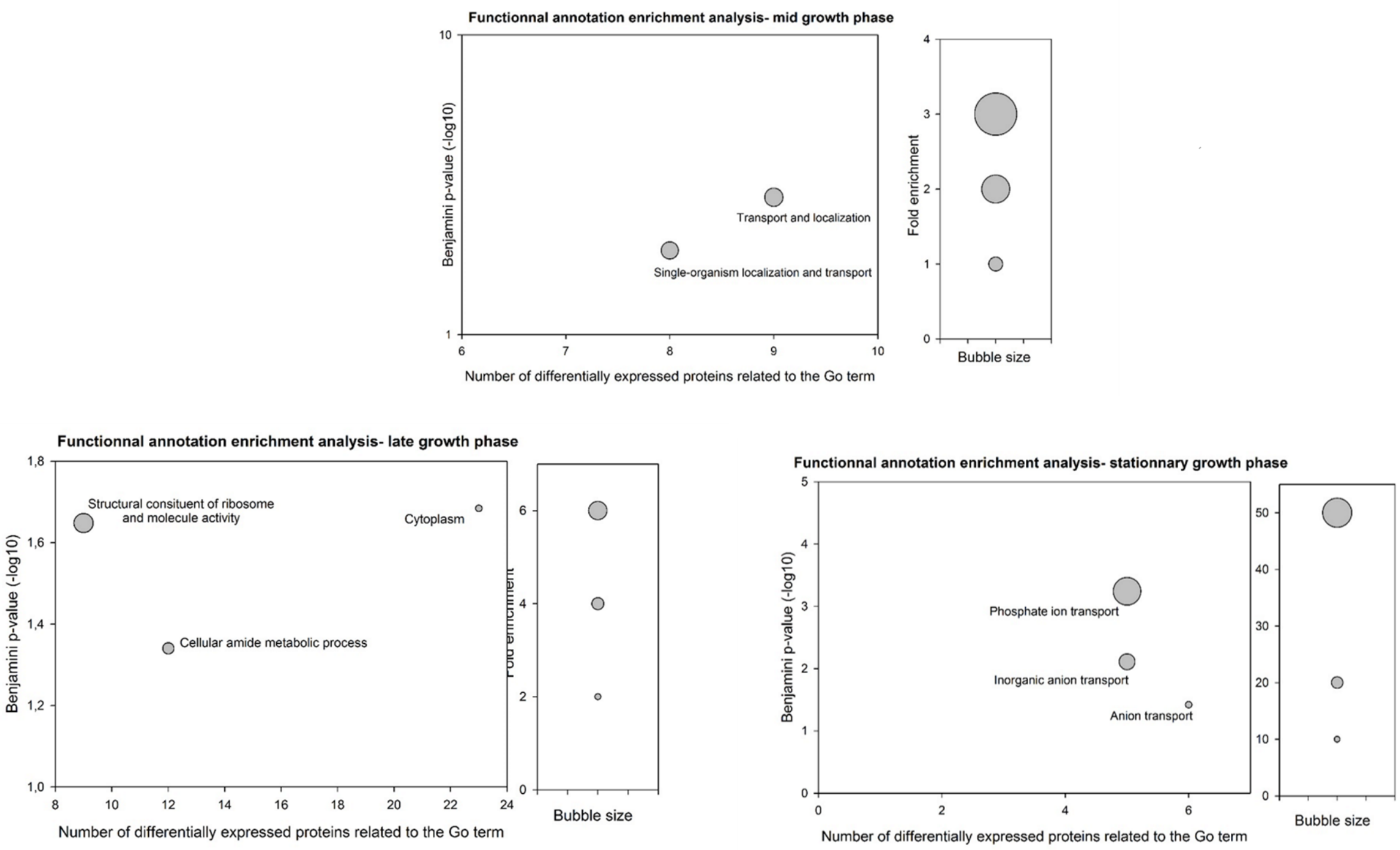


**Figure S4:** Functional annotation enrichment analysis of the DEGs computed using the scripts contained in the desktop version of DAVID (Ease v2.0) with stringent criteria: ease score < 0.1 and Benjamini *p* value < 0.05. The number of differentially produced proteins related to the GO term is represented on the x-axis and the Benjamini *p* value (-log10) on the y-axis. Bubble size is proportional to the fold enrichment of the GO term.

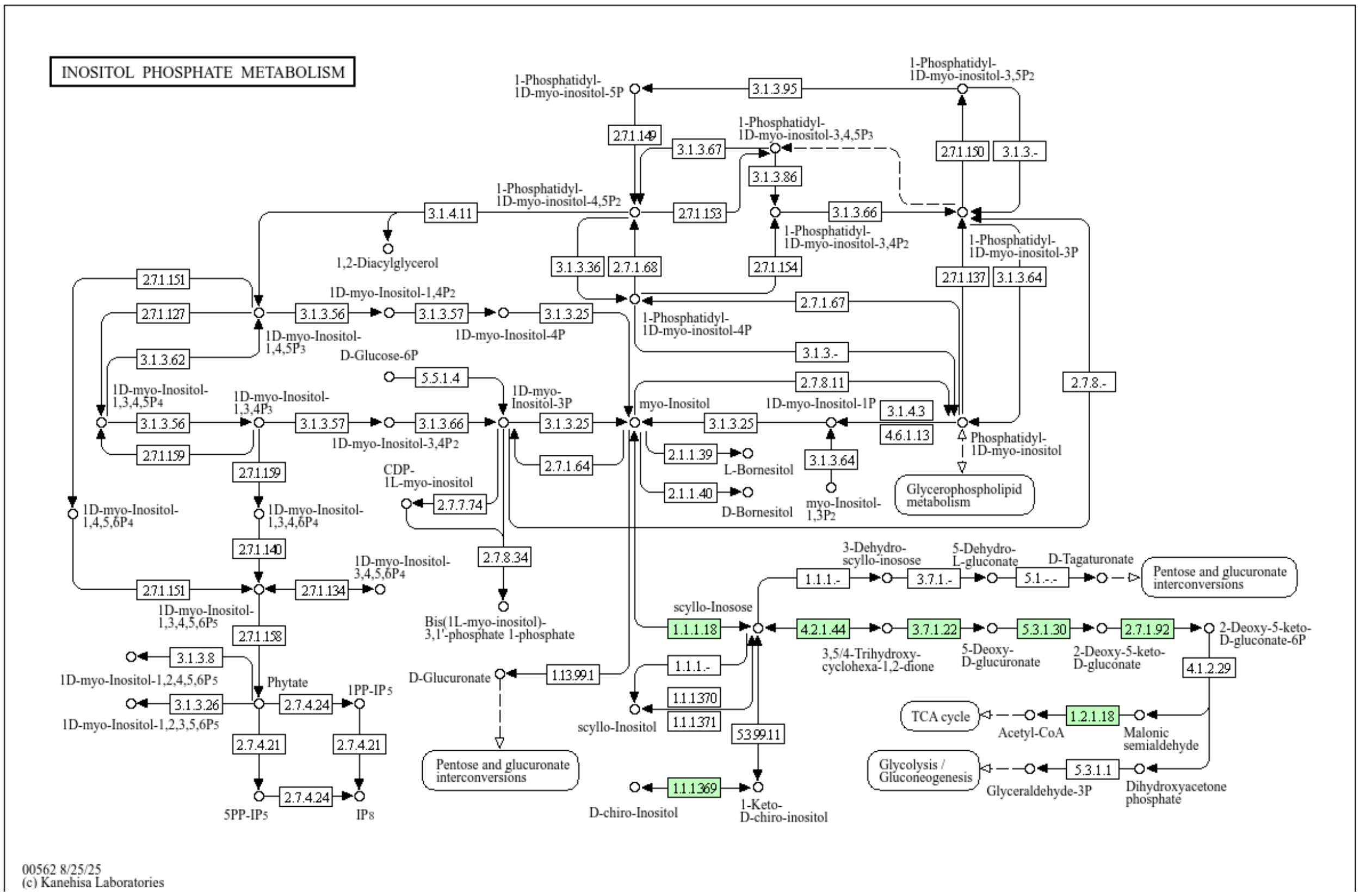


**Figure S5:** KEGG pathway map of inositol phosphate metabolism showing the mapping of genes (highlighted in green) from the gene cluster IGTS8_peg782-789 (Reproduced from Kanehisa Laboratories, Kanehisa et al., 2016).

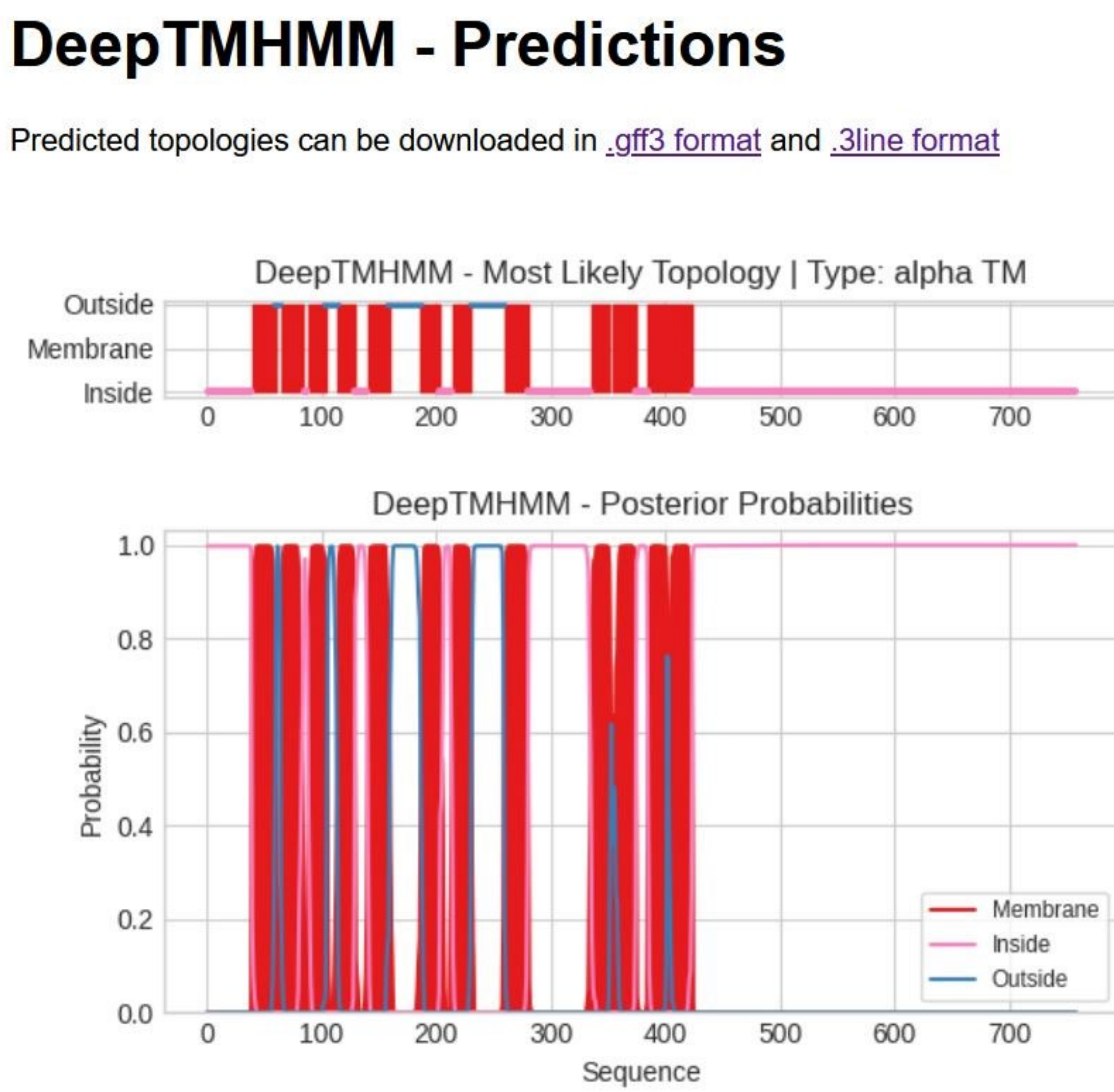


**Figure S6:** Predicted transmembrane regions in the protein sequence of the putative carbonic anhydrase IGTS8_peg3341. Protein sequence and predicted topologies are shown below.

```
>IGTS8_peg3341 Carbonic anhydrase (EC 4.2.1.1) 4842456:4844732 reverse
MW:78284
MSTQSVVHAQSSADESVVGDSSPPGGGSRRYLEQLIRYDVPASLVVFLVALPLSLGIAIA
SGAPIMAGLIAAIVGGVVAGAVGGSPLQVSGPAAGLTVVVAELIGKFGWKVTCLITVGAG
ILQIVFGLSRIARAALAIAPVVVHAMLAGIGITIALQQVHVLLGGSSASSALDNLRQLPA
QIGALHVEDLMIGLVVIATLLGWRYLPAGVRKVPAALVGIVLATLASLALPIDVERIALD
GSLLDSIGLPALPDGQWLGVATGILTIALIASVESLLSAVAVDKMHSGPRSNFDREMLGQ
GTANMVSGAIGGLPVTGVIVRSATNVEAGAKSRASSIMHGLWILLFSAALAGLVQQIPKS
ALAGLLIVIGIQLVKLAHIRLARRTGDLWVYGITVVGVVFLNLLEGVLIGLALAIALLVW
RVIRATVTAEELGDNDSGKWRVLVEGSCTFLVLPKLTSVLAGIPPRSDVTVELSVDFLDH
AVFDVIEEWARQYELAGGSVIIDEVGSVSMESATTGPPKRGAAVTDLRGLLAPWGSWQPQ
ASGRNLSSPPALRPILDGVSNYHRHRAEAIRPHMDQLRDGQNPDSLFLTCADSRVVPNII
TSSGPGDLFTVRNVGNLVPAGGRDTSVEAALAFGIDELGVSSVVVCGHSGCGAMQALLTN
GPSESGVVGEWLRHAETSLQAYTSGHPVARAAAEAGFSEVDQLGMVNVAVQLQTLERHPL
VGQARVDGRLRIAGLFFDIGSARVIEVTASGIGQLIET
```

**Predicted Topologies**

>IGTS8_peg3341 | TM

```
MSTQSVVHAQSSADESVVGDSSPPGGGSRRYLEQLIRYDVPASLVVFLVALPLSLGIAIASGAPIMAGLI
AAIVGGVVAGAVGGSPLQVSGPAAGLTVVVAELIGKFGWKVTCLITVGAGILQIVFGLSRIARAALAIAP
VVVHAMLAGIGITIALQQVHVLLGGSSASSALDNLRQLPAQIGALHVEDLMIGLVVIATLLGWRYLPAGV
RKVPAALVGIVLATLASLALPIDVERIALDGSLLDSIGLPALPDGQWLGVATGILTIALIASVESLLSAV
AVDKMHSGPRSNFDREMLGQGTANMVSGAIGGLPVTGVIVRSATNVEAGAKSRASSIMHGLWILLFSAAL
AGLVQQIPKSALAGLLIVIGIQLVKLAHIRLARRTGDLWVYGITVVGVVFLNLLEGVLIGLALAIALLVW
RVIRATVTAEELGDNDSGKWRVLVEGSCTFLVLPKLTSVLAGIPPRSDVTVELSVDFLDHAVFDVIEEWA
RQYELAGGSVIIDEVGSVSMESATTGPPKRGAAVTDLRGLLAPWGSWQPQASGRNLSSPPALRPILDGVS
NYHRHRAEAIRPHMDQLRDGQNPDSLFLTCADSRVVPNIITSSGPGDLFTVRNVGNLVPAGGRDTSVEAA
LAFGIDELGVSSVVVCGHSGCGAMQALLTNGPSESGVVGEWLRHAETSLQAYTSGHPVARAAAEAGFSEV
DQLGMVNVAVQLQTLERHPLVGQARVDGRLRIAGLFFDIGSARVIEVTASGIGQLIET
```

```
IIIIIIIIIIIIIIIIIIIIIIIIIIIIIIIIIIIIIIIMMMMMMMMMMMMMMMMMMMMMOOOOMMMMMM
MMMMMMMMMMMMMMIIIIMMMMMMMMMMMMMMMMOOOOOOOOOOMMMMMMMMMMMMMMMIIIIIIIIIII
MMMMMMMMMMMMMMMMMMMOOOOOOOOOOOOOOOOOOOOOOOOOOOMMMMMMMMMMMMMMMMMIIIIIII
IIIIMMMMMMMMMMMMMMMMOOOOOOOOOOOOOOOOOOOOOOOOOOOOOMMMMMMMMMMMMMMMMMMMMM
IIIIIIIIIIIIIIIIIIIIIIIIIIIIIIIIIIIIIIIIIIIIIIIIIIIIIIIMMMMMMMMMMMMMMM
MOOMMMMMMMMMMMMMMMMMMMMMIIIIIIIIIIMMMMMMMMMMMMMMMMMOMMMMMMMMMMMMMMMMMM
MMMIIIIIIIIIIIIIIIIIIIIIIIIIIIIIIIIIIIIIIIIIIIIIIIIIIIIIIIIIIIIIIIIIII
IIIIIIIIIIIIIIIIIIIIIIIIIIIIIIIIIIIIIIIIIIIIIIIIIIIIIIIIIIIIIIIIIIIIII
IIIIIIIIIIIIIIIIIIIIIIIIIIIIIIIIIIIIIIIIIIIIIIIIIIIIIIIIIIIIIIIIIIIIII
IIIIIIIIIIIIIIIIIIIIIIIIIIIIIIIIIIIIIIIIIIIIIIIIIIIIIIIIIIIIIIIIIIIIII
IIIIIIIIIIIIIIIIIIIIIIIIIIIIIIIIIIIIIIIIIIIIIIIIIIIIIIIIII
```

##gff-version 3
# IGTS8_peg3341 Length: 758
# IGTS8_peg3341 Number of predicted TMRs: 12
IGTS8_peg3341 inside 1 39
IGTS8_peg3341 TMhelix 40 60
IGTS8_peg3341 outside 61 64
IGTS8_peg3341 TMhelix 65 84
IGTS8_peg3341 inside 85 88
IGTS8_peg3341 TMhelix 89 104
IGTS8_peg3341 outside 105 114
IGTS8_peg3341 TMhelix 115 129
IGTS8_peg3341 inside 130 140
IGTS8_peg3341 TMhelix 141 159
IGTS8_peg3341 outside 160 186
IGTS8_peg3341 TMhelix 187 203
IGTS8_peg3341 inside 204 214
IGTS8_peg3341 TMhelix 215 230
IGTS8_peg3341 outside 231 259

IGTS8_peg3341 TMhelix 260 280
IGTS8_peg3341 inside 281 335
IGTS8_peg3341 TMhelix 336 351
IGTS8_peg3341 outside 352 353
IGTS8_peg3341 TMhelix 354 374
IGTS8_peg3341 inside 375 384
IGTS8_peg3341 TMhelix 385 401
IGTS8_peg3341 outside 402 402
IGTS8_peg3341 TMhelix 403 423
IGTS8_peg3341 inside 424 758

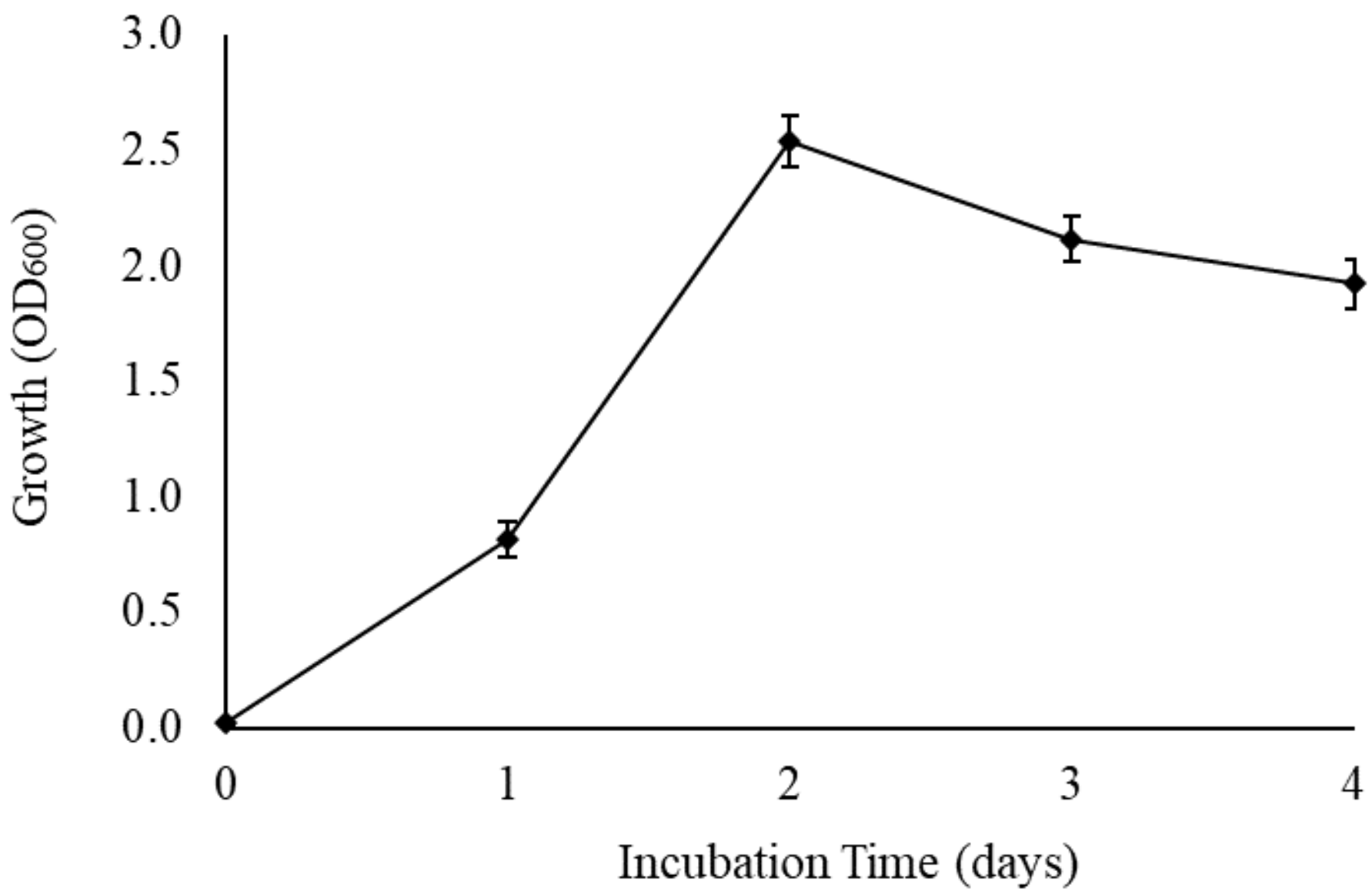


**Figure S7:** Growth of the strain IGTS8 in CDM containing acetonitrile (95.2 mM) as the sole carbon source and $MgSO_4$ as the sulfur source.

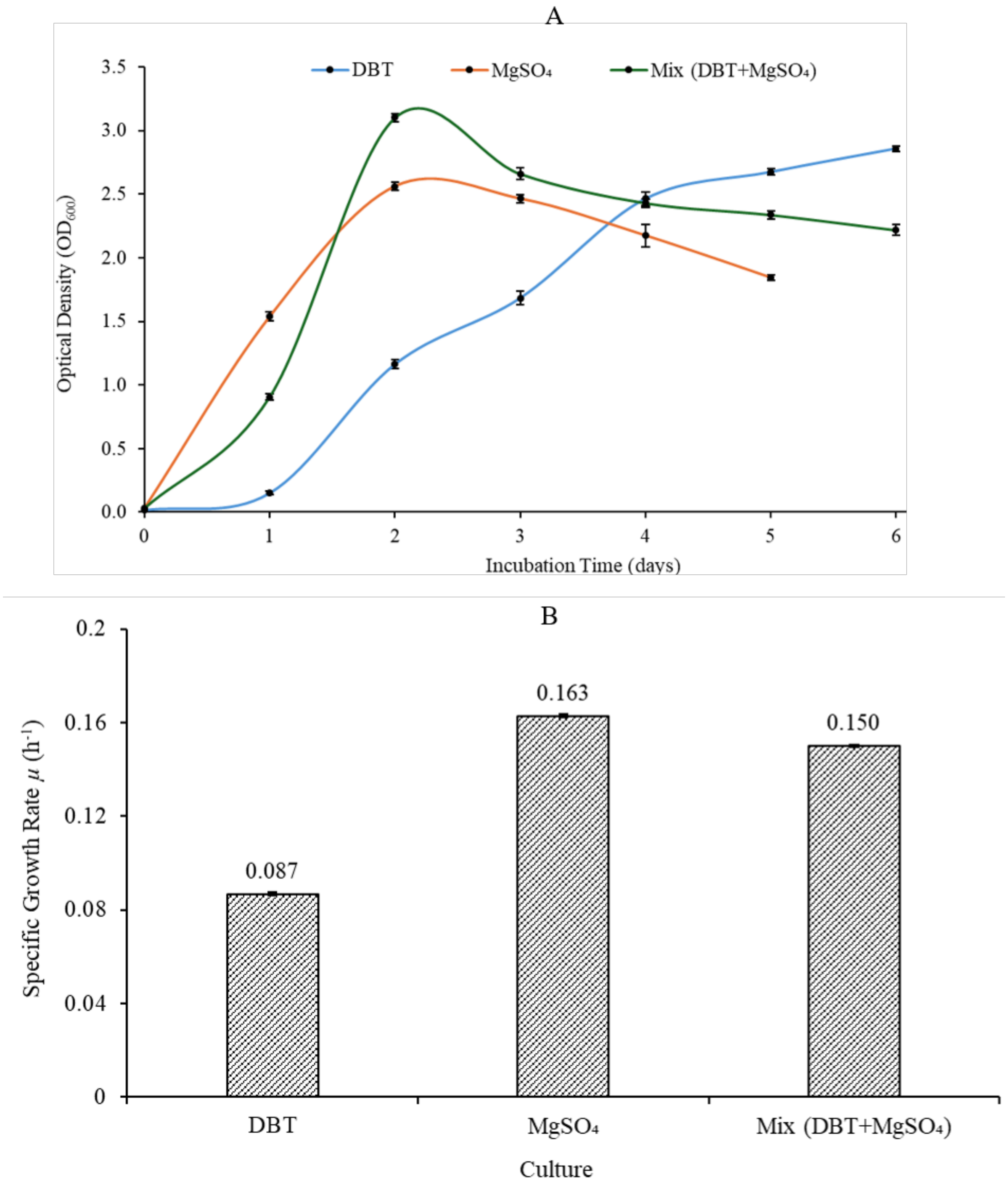


**Figure S8:** Growth profiles (A) and specific growth rates (B) of *R. qingshengii* IGTS8 cultures grown on 0.1 mM of either $MgSO_4$, DBT, or a mixture of both as the sulfur source and 10 mM glucose + 17.14 mM ethanol as the carbon source. All values are average of three biological replicates.

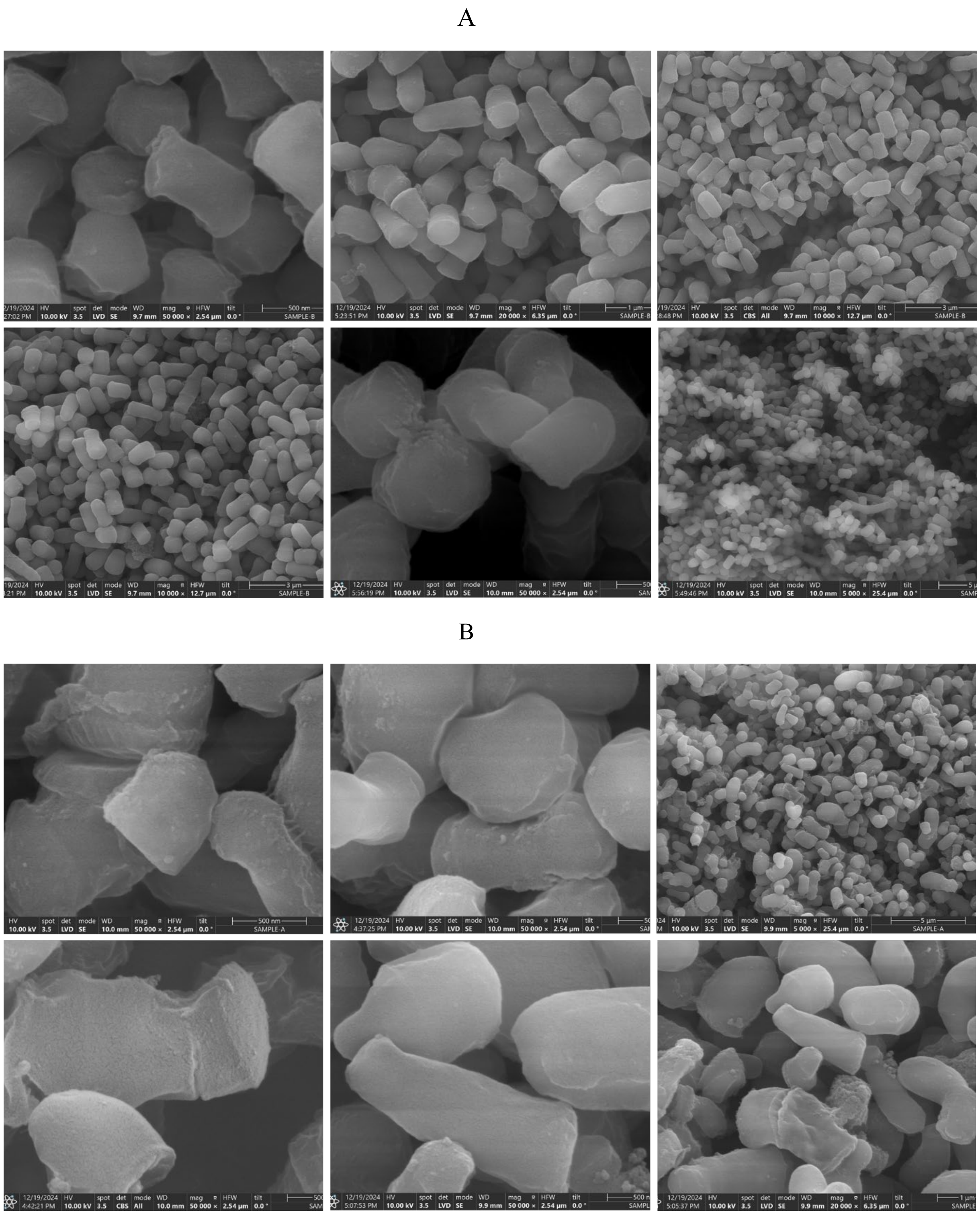


**Figure S9**: Scanning electron microscopy images of cultures of the strain IGTS8 grown in CDM for 42 h with either $MgSO_4$ (A) or DBT (B) as the sulfur source and glucose + ethanol as the carbon source.

**References**

Kanehisa M, Sato Y, Morishima K. 2016. BlastKOALA and GhostKOALA: KEGG tools for functional characterization of genome and metagenome sequences. J Mol Biol 428:726–731. doi: 10.1016/j.jmb.2015.11.006.
